# Enhancement of β-Lactam-Mediated Killing of Gram-Negative Bacteria by Lysine Hydrochloride

**DOI:** 10.1128/spectrum.01198-23

**Published:** 2023-06-13

**Authors:** Shouqiang Hong, Shaopeng Su, Qiong Gao, Miaomiao Chen, Lisheng Xiao, Runbo Cui, Yinli Guo, Yunxin Xue, Dai Wang, Jianjun Niu, Haihui Huang, Xilin Zhao

**Affiliations:** a State Key Laboratory of Vaccines for Infectious Diseases, Xiang An Biomedicine Laboratory, Department of Laboratory Medicine, School of Public Health, Xiamen University, Xiamen, Fujian Province, China; b Institute of Antibiotics, Huashan Hospital, Fudan University, Shanghai, China; c Center of Clinical Laboratory, Zhongshan Hospital, School of Medicine, Xiamen University, Xiamen, Fujian Province, China; South China Sea Institute of Oceanology

**Keywords:** lysine hydrochloride, β-lactam antibiotics, enhanced killing, arginine hydrochloride, lipopolysaccharide, reactive oxygen species (ROS), FtsH protease

## Abstract

Widespread bacterial resistance among Gram-negative bacteria is rapidly depleting our antimicrobial arsenal. Adjuvants that enhance the bactericidal activity of existing antibiotics provide a way to alleviate the resistance crisis, as new antimicrobials are becoming increasingly difficult to develop. The present work with Escherichia coli revealed that neutralized lysine (lysine hydrochloride) enhances the bactericidal activity of β-lactams in addition to increasing bacteriostatic activity. When combined, lysine hydrochloride and β-lactam increased expression of genes involved in the tricarboxylic acid (TCA) cycle and raised reactive oxygen species (ROS) levels; as expected, agents known to mitigate bactericidal effects of ROS reduced lethality from the combination treatment. Lysine hydrochloride had no enhancing effect on the lethal action of fluoroquinolones or aminoglycosides. Characterization of a tolerant mutant indicated involvement of the FtsH/HflkC membrane-embedded protease complex in lethality enhancement. The tolerant mutant, which carried a V86F substitution in FtsH, exhibited decreased lipopolysaccharide levels, reduced expression of TCA cycle genes, and reduced levels of ROS. Lethality enhancement by lysine hydrochloride was abolished by treating cultures with Ca^2+^ or Mg^2+^, cations known to stabilize the outer membrane. These data, plus damage observed by scanning electron microscopy, indicate that lysine stimulates β-lactam lethality by disrupting the outer membrane. Lethality enhancement of β-lactams by lysine hydrochloride was also observed with Acinetobacter baumannii and Pseudomonas aeruginosa, thereby suggesting that the phenomenon is common among Gram-negative bacteria. Arginine hydrochloride behaved in a similar way. Overall, the combination of lysine or arginine hydrochloride and β-lactam offers a new way to increase β-lactam lethality with Gram-negative pathogens.

**IMPORTANCE** Antibiotic resistance among Gram-negative pathogens is a serious medical problem. The present work describes a new study in which a nontoxic nutrient increases the lethal action of clinically important β-lactams. Elevated lethality is expected to reduce the emergence of resistant mutants. The effects were observed with significant pathogens (Escherichia coli, Acinetobacter baumannii, and Pseudomonas aeruginosa), indicating widespread applicability. Examination of tolerant mutants and biochemical measurements revealed involvement of endogenous reactive oxygen species in response to outer membrane perturbation. These lysine hydrochloride–β-lactam data support the hypothesis that lethal stressors can stimulate the accumulation of ROS. Genetic and biochemical work also revealed how an alteration in a membrane protease, FtsH, abolishes lysine stimulation of β-lactam lethality. Overall, the work presents a method for antimicrobial enhancement that should be safe, easy to administer, and likely to apply to other nutrients, such as arginine.

## INTRODUCTION

Gram-negative bacteria, such as Escherichia coli, Klebsiella pneumoniae, Acinetobacter baumannii, and Pseudomonas aeruginosa, are major causes of both community-acquired and nosocomial infections (1–4). The high prevalence of antibiotic resistance among these pathogens has rendered treatment with existing agents problematic. Since novel antimicrobials have been difficult to identify and develop (5–8), new strategies to control these pathogens are urgently needed. One approach is to identify ways to enhance antimicrobial lethality, because that will reduce the emergence of new resistant mutants by lowering the bacterial burden during infection and by killing resistant mutant subpopulations (9). Common organic substances in the human body, such as amino acids, sugars, and their metabolites, have low toxicity and thus have been examined as potential lethality enhancers. Examples include lysine, glutamate, serine, valine, glutamine, glucose, mannitol, fructose, and pyruvic acid (10–18). The present work focused on l-lysine, an amino acid essential for humans.

When added to cultures, lysine enhanced the bactericidal activity of aminoglycosides by elevating proton motive force and increasing permeability of cell membranes; that led to intracellular accumulation of reactive oxygen species (ROS) and bacterial death (19). Whether lysine has a direct interaction with cells to enhance antimicrobial lethality is unclear, because in solution it has a high pH that alone may cause nonspecific alkaline-mediated bactericidal activity, a possibility that has not been addressed previously. To determine whether lysine has a specific killing mechanism rather than an indirect pH effect, we examined neutralized lysine (lysine hydrochloride [lysine HCl]) to rule out alkaline-mediated killing. Surprisingly, lysine HCl enhanced activity of β-lactams, but not that of fluoroquinolones or aminoglycosides. Examination of tolerant mutants and biochemical measurements showed that lysine hydrochloride interacts with lipopolysaccharide (LPS) and destabilizes the outer membrane. It also promotes β-lactam-stimulated intracellular accumulation of ROS, thereby solidifying the hypothesis that lethal stressors can act by stimulating the accumulation of ROS. Overall, increasing the binding efficiency of lysine or its derivatives to LPS represents a novel approach for enhancing β-lactam lethality.

## RESULTS

### Effect of lysine hydrochloride on the bacteriostatic action of antimicrobials with E. coli.

We began by examining the effect of lysine hydrochloride (lysine HCl) on inhibition of growth (the bacteriostatic and bactericidal actions of antimicrobials differ mechanistically [20, 21]). With a laboratory strain of E. coli (BW25113), the presence of lysine hydrochloride, at half its MIC, decreased the MICs of ampicillin, vancomycin, colistin, and erythromycin 2- to 8-fold, but lysine hydrochloride showed little effect on the MICs of kanamycin and ciprofloxacin (Table 1). We then performed a checkerboard analysis (22) to determine the fractional inhibitory concentration index (FICI) for lysine hydrochloride when combined with several widely used antimicrobials. By this test, lysine hydrochloride had additive effects with β-lactams (ampicillin, ceftriaxone, and meropenem), colistin, vancomycin, and erythromycin; however, lysine hydrochloride was indifferent to kanamycin and ciprofloxacin (Table 1). Thus, lysine hydrochloride slightly enhances bacteriostatic susceptibility of antimicrobials whose targets are the cell wall or cell membrane and, in one case, protein synthesis (erythromycin, a macrolide).

**TABLE 1:** Effect of neutralized lysine on antimicrobial susceptibility of E. coli

Agent	MIC^*a*^			
	Alone	Combination^*b*^	FICI	Interpretation
Lysine hydrochloride	1			
Lysine	0.06			
Ampicillin	4	1	0.75	Additive
Ceftriaxone	0.04	0.01	0.75	Additive
Meropenem	0.04	0.01	0.75	Additive
Kanamycin	4	4	1.5	Indifferent
Ciprofloxacin	0.02	0.02	1.5	Indifferent
Vancomycin	500	62.5	0.625	Additive
Erythromycin	48	24	1	Additive
Colistin	0.6	0.125	0.75	Additive

a MICs are in micrograms per milliliter except for those of lysine hydrochloride and lysine, which are molar concentrations.

b MIC when the antimicrobial was tested in combination with 0.5 M lysine hydrochloride.

### Effect of lysine hydrochloride on bactericidal action of β-lactams.

**FIG 1:**
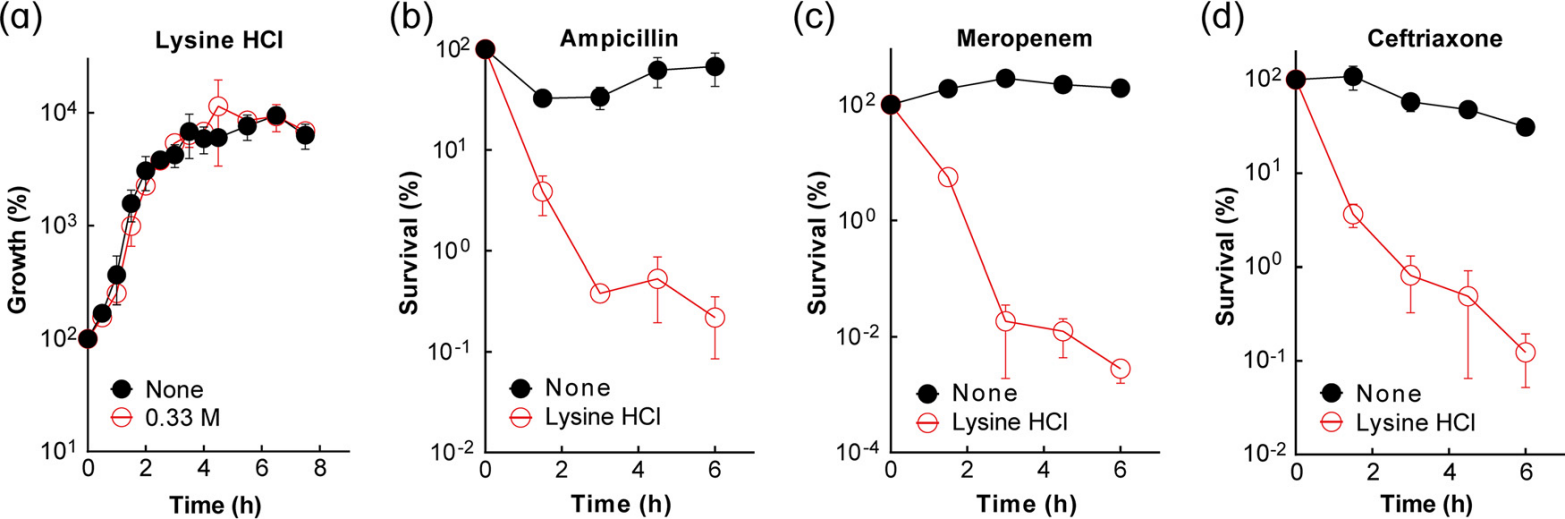
Enhancement of β-lactam killing by lysine hydrochloride. (a) Addition of lysine hydrochloride has little effect on bacterial growth. Exponentially growing cultures of wild-type E. coli (BW25113; strain 1) were treated or not with 0.33 M (1/3× MIC) lysine hydrochloride for the indicated times before samples were taken for determination of CFU. (b to d) Lysine hydrochloride-enhanced β-lactam-mediated killing with 3 β-lactams. Exponentially growing cultures of E. coli were treated with 1× MIC ampicillin (b), 2× MIC meropenem (c), or 2× MIC ceftriaxone (d) in the presence/absence of 0.33 M lysine hydrochloride for the indicated times, after which CFU was determined relative to the culture at the time of lysine hydrochloride addition. Data represent the means from 3 biological replicates; error bars indicate standard deviations.

We next determined whether lysine or neutralized lysine alone is bactericidal. Lysine killed E. coli rapidly and extensively, but lysine hydrochloride did not (see Fig. S1 in the supplemental material). Since lysine likely kills bacteria through medium alkalinization, it was not studied further. We next examined the effect of subinhibitory concentrations of lysine hydrochloride on the bactericidal activity of the β-lactam ampicillin, since we observed an additive effect on inhibition of growth when the two compounds were combined (Table 1). Since growth inhibition by lysine hydrochloride (or any compound) itself would inhibit β-lactam killing (β-lactam killing is growth dependent), we first defined subinhibitory/noninhibitory concentrations of lysine hydrochloride that can be used to evaluate β-lactam killing without interference from nonspecific, growth inhibition-mediated reduction of killing. At 0.5 M lysine hydrochloride, culture growth was reduced (Fig. S2a), but at 0.33, 0.25, or 0.125 M, no effect was observed (Fig. 1a and Fig. S2a). When lysine hydrochloride was added to exponentially growing cultures at 0.33 M (1/3× MIC) in the presence of ampicillin (1× MIC), meropenem (2× MIC), or ceftriaxone (2× MIC) for various times, survival dropped sharply (1,000- to 10,000-fold in 3 h). Cultures treated only with β-lactam at these low concentrations showed little killing (Fig. 1b to d). Lysine hydrochloride, alone at 0.33 M, showed no effect on either growth or survival (Fig. 1a). Lysine hydrochloride at 0.25 and 0.125 M also stimulated β-lactam-mediated killing, particularly for ampicillin (Fig. S2); further reduction of lysine hydrochloride concentration (16.7 or 0.333 mM) failed to show any killing enhancement even when ampicillin concentration was increased to 8× MIC (Fig. S3). We conclude that lysine hydrochloride enhances the lethal activity of β-lactams. Since lysine hydrochloride stimulation of bacterial killing was not observed with two other major bactericidal antimicrobial classes, aminoglycosides and fluoroquinolones (Fig. S4), we focused mechanism studies on β-lactams.

### Lysine hydrochloride functions exogenously, possibly by altering outer membrane integrity.

**FIG 2:**
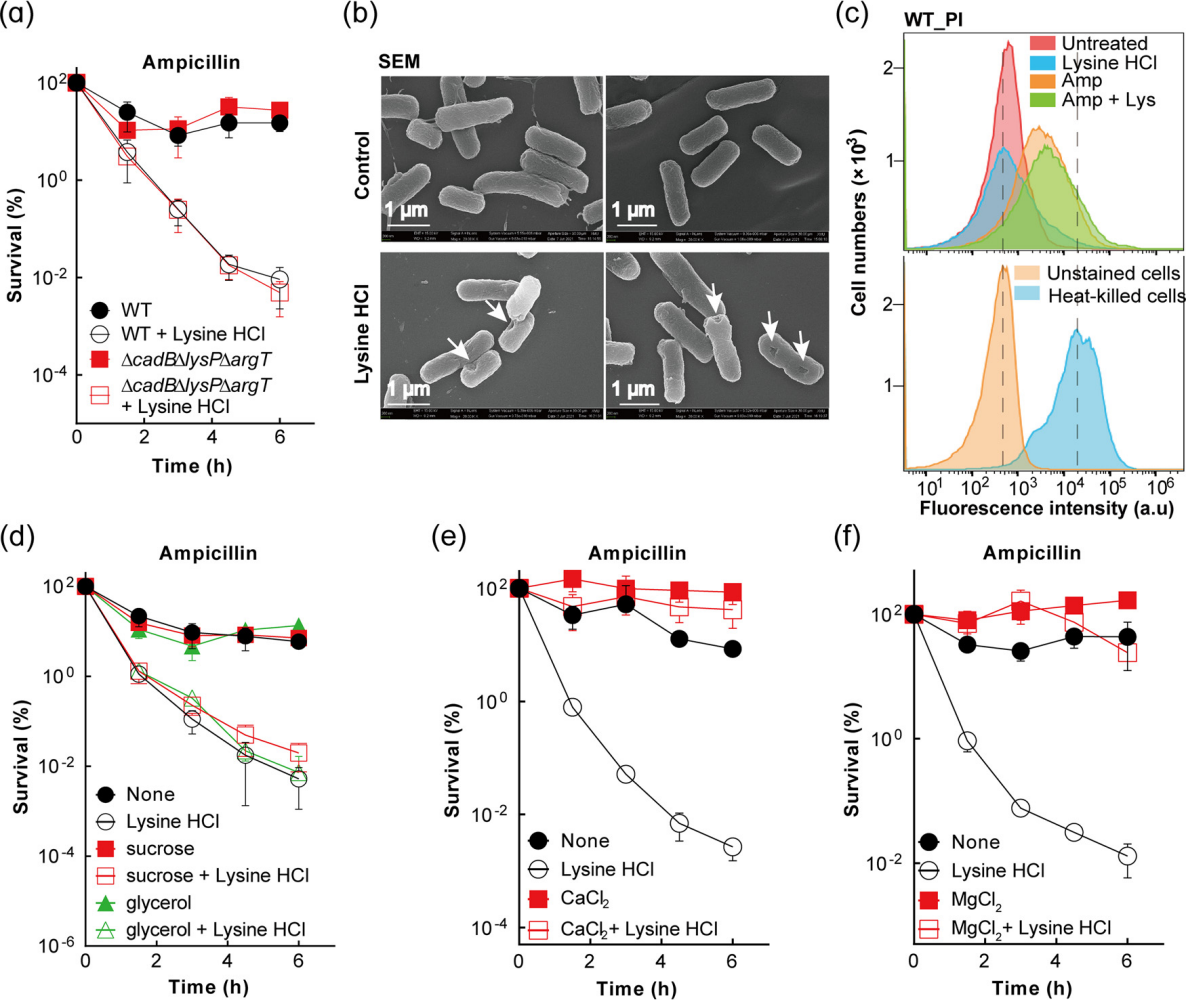
Lysine hydrochloride enhancement of β-lactam-mediated killing through interruption of the outer membrane. (a) Cellular uptake of lysine hydrochloride is not required for enhancement of ampicillin-mediated killing. Exponentially growing cultures of wild-type E. coli (strain 1) and the Δ*cadB* Δ*lysP* Δ*argT* triple mutant (strain 5) were treated with 1× MIC ampicillin in the presence/absence of 0.33 M (1/3× MIC) lysine hydrochloride for the indicated times before percent survival was determined as for Fig. 1. (b) Lysine damages the bacterial cell surface. Exponentially growing cultures of wild-type E. coli were treated or not with 0.33 M lysine hydrochloride for 1.5 h before they were subjected to scanning electron microscopy. Arrows point to areas of membrane damage. Similar data were obtained from 3 biological replicates, with one representative data set being presented here. (c) Restriction of propidium iodide (PI) entry unaffected by treatment with lysine hydrochloride. Exponentially growing cultures of wild-type E. coli were pretreated with PI for 20 min and then treated or not (control) with 0.33 M lysine hydrochloride in the presence or absence of ampicillin for 1 h before cells were subjected to flow cytometry analysis of the PI-stained cell population. Unstained cells and heat-killed cells were used as negative and positive controls. Similar data were obtained from 3 biological replicates, with one representative data set being presented here. a.u, arbitrary units. (d) Maintenance of osmotic pressure failed to eliminate lysine hydrochloride-mediated enhancement of ampicillin-mediated killing. Exponentially growing cultures of wild-type E. coli were treated with 1× MIC ampicillin alone or in combination with 0.33 M lysine hydrochloride, 10% sucrose, 0.5% glycerol, or lysine hydrochloride plus sucrose or glycerol for the indicated times, after which CFU were determined at the indicated times. (e and f) Divalent cations eliminated lysine hydrochloride enhancement of ampicillin lethality. Exponentially growing cultures of wild-type E. coli were treated with 1× MIC ampicillin with or without 0.33 M lysine hydrochloride in the presence or absence of 20 mM CaCl_2_ (e) or 62.5 mM MgCl_2_ (f). CFU were determined at the indicated times. Data represent the means from 3 biological replicates; error bars indicate standard deviations.

To determine whether entering the cell is necessary for enhancement of ampicillin-mediated killing, we measured killing with Δ*cadB*, Δ*lysP*, and Δ*argT* lysine transport mutants individually and as a Δ*cadB* Δ*lysP* Δ*argT* triple mutant. These mutants, which exhibited no effect on MIC of ampicillin or lysine hydrochloride (Table S1), showed little impact on lysine hydrochloride-mediated enhancement of killing (Fig. 2a and Fig. S5). These mutations also had little or no effect on the small bactericidal effect of ampicillin alone (Fig. 2a and Fig. S5). Thus, entry into the cell appears to be unnecessary for stimulation of lethality.

When we used scanning electron microscopy to examine a possible interaction of lysine hydrochloride with extracellular components of E. coli, we found that the cell surface, either the outer membrane or the cell wall, appeared damaged (Fig. 2b and Fig. S6). Inner membrane integrity was not compromised, because propidium iodide staining of E. coli cultures treated with 0.33 M lysine hydrochloride either alone or in combination with ampicillin, followed by flow cytometry analysis, showed no significant increase in membrane permeability compared with respective control samples (e.g., untreated cells or cells treated with ampicillin alone [Fig. 2c]).

To determine whether weakening of the cell wall contributed to lysine hydrochloride stimulation of killing, we added sucrose or glycerol to the culture medium to maintain osmotic pressure sufficient to mitigate killing from cell wall rupture and high intracellular osmotic pressure. Adding 10% sucrose or 0.5% glycerol to growth medium showed little effect on lysine hydrochloride-mediated enhancement of killing (Fig. 2d). Thus, lysine hydrochloride is unlikely to stimulate killing by directly weakening the cell wall. That left the outer membrane as a potential target. Since divalent cations (e.g., CaCl_2_, MgCl_2_) are known to stabilize the outer membrane (23–26), we added these cations at noninhibitory concentrations (Table S2) to cultures: they reduced enhancement of ampicillin lethality due to lysine hydrochloride (Fig. 2e and f). Collectively, these data are consistent with lysine hydrochloride enhancing β-lactam killing by destabilizing the outer membrane of E. coli.

### Enrichment and characterization of mutants tolerant to lysine hydrochloride enhancement of ampicillin lethality.

**FIG 3:**
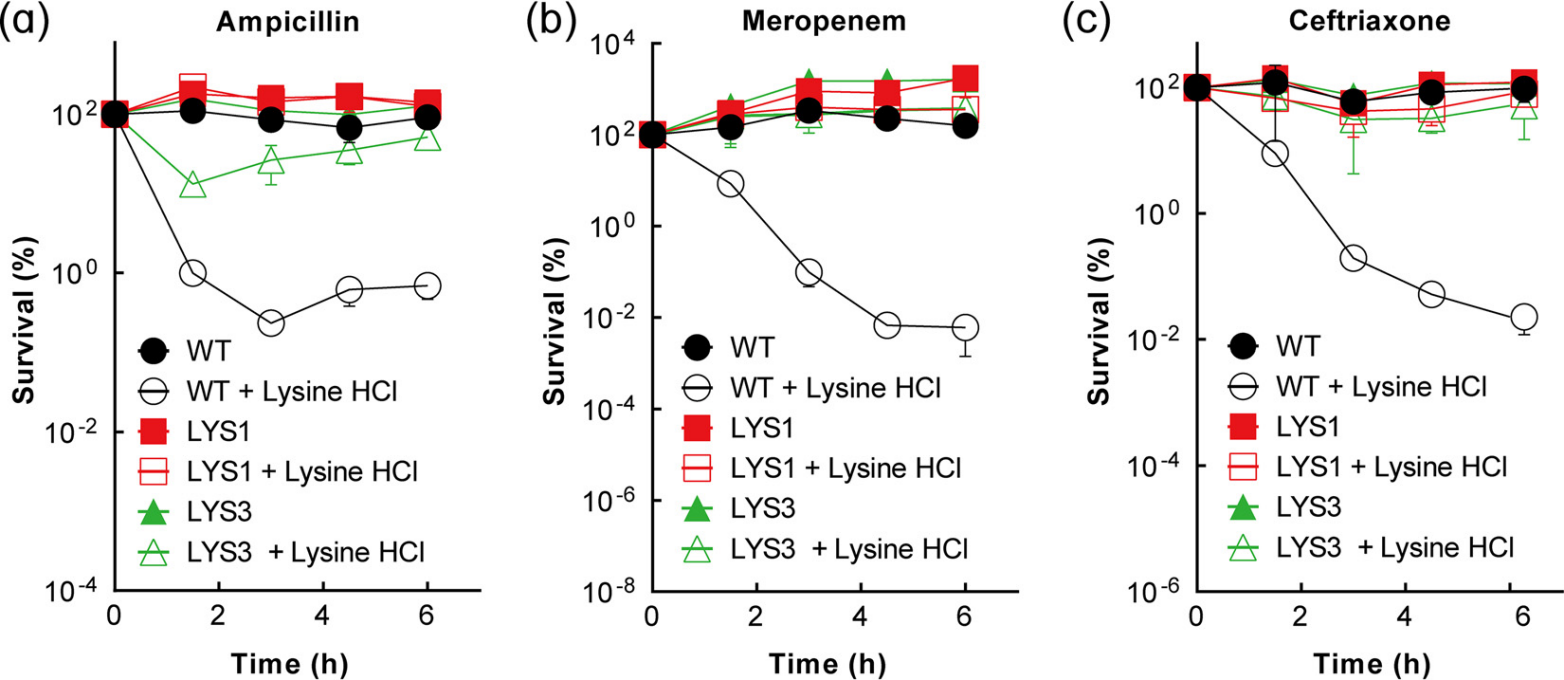
Phenotype properties of mutants tolerant to lysine hydrochloride enhancement of β-lactam lethality. Wild-type (WT; strain 1), LYS1 (strain 6), and LYS3 (strain 7) E. coli cultures were grown to exponential phase and then treated with 1× MIC ampicillin (a), 2× MIC meropenem (b), or 2× MIC ceftriaxone (c) in the presence/absence of 1/3× MIC lysine hydrochloride for the indicated times. Percent survival was determined by CFU measurement relative to a sample taken at the time of antimicrobial addition. Data represent the means from 3 biological replicates; error bars indicate standard deviations.

To gain insight into the mechanism underlying lethality enhancement by lysine hydrochloride, we enriched E. coli cultures for mutants tolerant to lysine-enhanced killing by ampicillin. Exponentially growing, wild-type (WT) cultures were treated with ampicillin (1× MIC) plus 0.33 M (1/3× MIC) lysine hydrochloride for 4.5 h, which led to a >100-fold drop in survival (Fig. 1b). Surviving cells were cultured, and the challenge with lysine hydrochloride and ampicillin was repeated. After 10 rounds of treatment, tolerant mutants were obtained. Two mutants, designated LYS1 and LYS3, were examined. In a comparison with wild-type cells, no significant change in MIC of either mutant occurred (Tables S1 and S2), indicating that the loss of lethality was not caused by a change in the initial drug-target interaction that blocks bacterial growth. The two mutations raised bacterial survival >100-fold during a 6-h treatment with 1× MIC ampicillin plus 1/3× MIC lysine hydrochloride (Fig. 3a) and >1,000-fold with meropenem (2× MIC) or ceftriaxone (2× MIC) in the presence of 1/3× MIC lysine hydrochloride (Fig. 3b and c).

**FIG 4:**
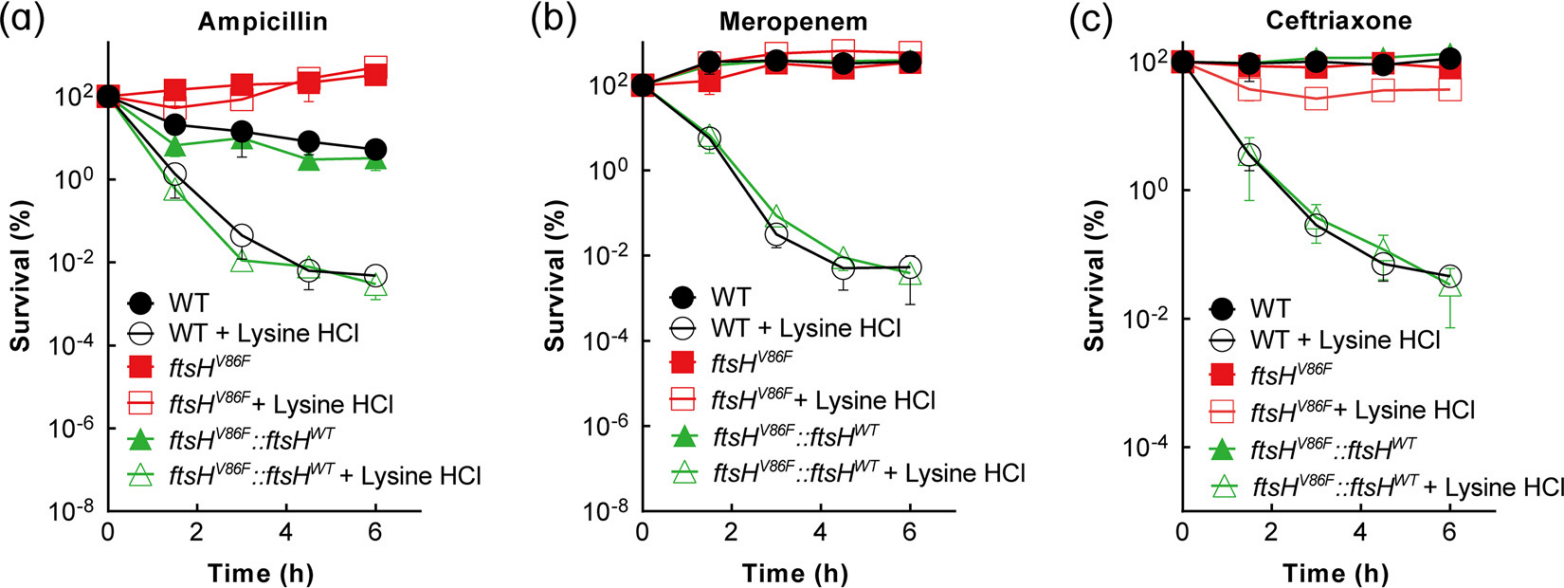
Mutation of *ftsH* confers tolerance to lysine hydrochloride enhancement of β-lactam lethality. Exponentially growing cultures of wild-type E. coli (strain 1), an *ftsH* V86F mutant (strain 8), and a strain in which *ftsH* V86F was replaced with wild-type *ftsH* (strain 9) were treated with 1× MIC ampicillin (a), 2× MIC meropenem (b), or 2× MIC ceftriaxone (c) in the presence or absence of 1/3× MIC lysine hydrochloride for the indicated times. Percent survival was determined at the indicated times by CFU determination relative to CFU at the time of drug addition. Data represent the means from 3 biological replicates; error bars indicate standard deviations.

Whole-genome sequencing of LYS1 and LYS3 revealed that both mutants harbored a missense mutation (V86F) in *ftsH*, which encodes an ATP-dependent metalloprotein of the FtsH/HflkC membrane protein degradation complex. LYS3 also harbored a point mutation 13 nucleotides upstream from the transcription initiation site of *gntK*, which encodes a kinase involved in the d-gluconate degradation pathway (Table 2). Since the *gntK* mutation might affect transcription, we examined the bactericidal phenotypes of a Δ*gntK* mutant and a *gntK* overexpression strain (WT-pBAD18-*gntK*). Neither strain exhibited an effect on tolerance to lysine hydrochloride-ampicillin-mediated killing (Fig. S7). A point mutation (V86F) in *ftsH* was also reconstructed in the parental, wild-type strain, followed by growth and bacterial killing assays. The reconstructed strain showed the same tolerant phenotype to the lysine hydrochloride–β-lactam combination as seen with the LYS1 and LYS3 mutants (Fig. 4). No effect on growth inhibition was seen with the reconstructed *ftsH* V86F mutant (Fig. S8a). The susceptibility of the *ftsH* V86F mutant to the lysine hydrochloride-ampicillin combination was also restored to the wild-type level by using CRISPR genome editing to replace the *ftsH* mutant allele with the homologous wild-type gene (Fig. 4). The *ftsH* V86F mutant showed no effect on ampicillin lethality in the absence of lysine hydrochloride (Fig. S8b). We conclude that a single point mutation in *ftsH* confers tolerance specifically to the enhancement of β-lactam-mediated killing by lysine hydrochloride. Because the *ftsH* V86F, the LYS1, and the LYS3 mutants exhibited similar tolerance phenotypes, the reconstructed *ftsH* V86F mutant was used in subsequent studies.

**TABLE 2:** Genetic changes detected in mutants tolerant to killing by the lysine hydrochloride-ampicillin combination

Mutant strain	Gene	Base change	Amino acid change	Gene product
LYS1	*ftsH*	G256T	Val86Phe	ATP-dependent zinc metalloprotease FtsH
LYS3	*ftsH*	G256T	Val86Phe	ATP-dependent zinc metalloprotease FtsH
	*gntK*	A +^*a*^ 13G		d-Gluconate kinase, thermostable

a +, upstream of transcription start site.

### Decreased outer membrane LPS content mediated by the *ftsH* V86F substitution.

**FIG 5:**
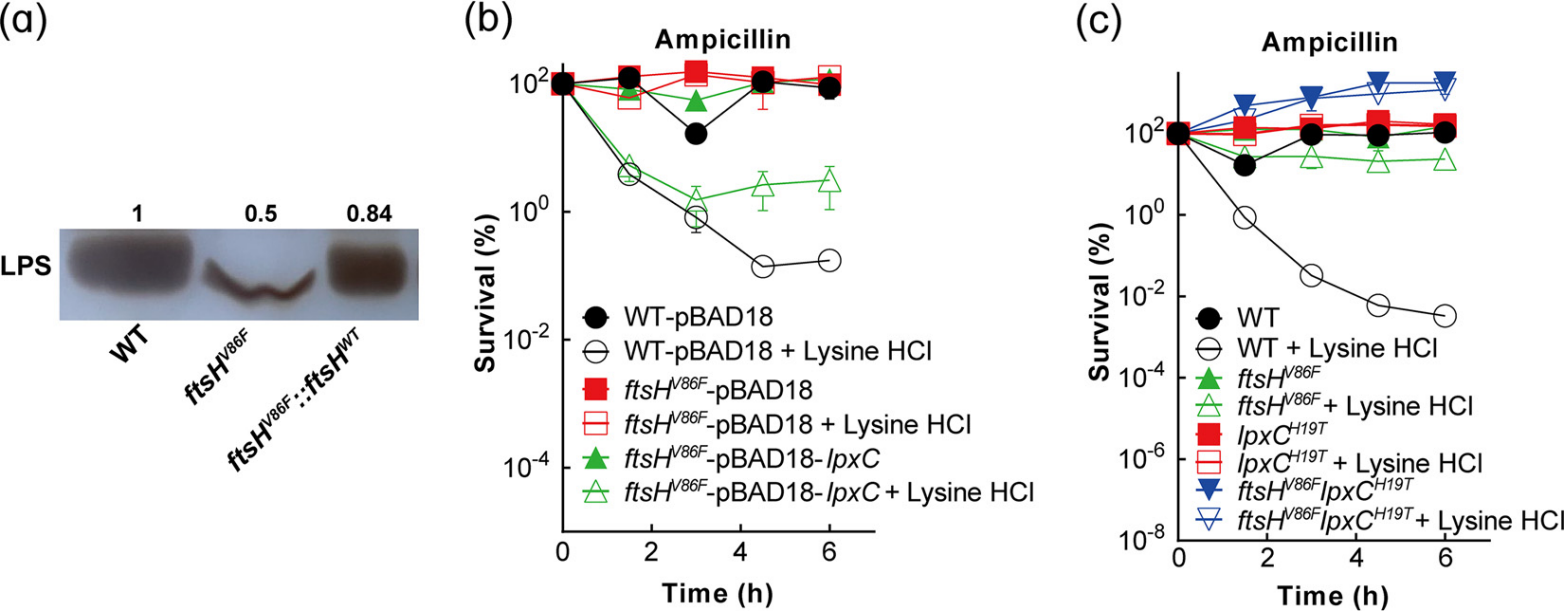
Reduced LPS level associated with tolerance to lysine hydrochloride enhancement of β-lactam-mediated lethality. (a) Reduction of LPS content in *ftsH* V86F mutant. Exponentially growing cultures (OD_600_ = 0.3) of wild-type E. coli (strain 1), an *ftsH* V86F mutant (strain 8), and an *ftsH^V86F^*::*ftsH^WT^* (strain 9) were grown for 1.5 h, after which samples were collected for LPS extraction. LPS levels were detected by silver staining after SDS-PAGE. (b) Overexpression of *lpxC* reversed *ftsH* V86F-mediated tolerance to enhancement of β-lactam lethality. Exponentially growing cultures of E. coli wild-type strain harboring pBAD18 (strain 11), an *ftsH* V86F mutant harboring pBAD18 (strain 13), and an *ftsH* V86F mutant harboring pBAD18-*lpxC* (strain 14) were treated with ampicillin alone or a lysine hydrochloride-ampicillin combination for the indicated times before samples were taken, diluted, and plated for percent survival determination. (c) Reduction-of-function *lpxC* (LPS) mutation increased lysine hydrochloride tolerance for β-lactams when combined with the *ftsH* V86F mutation. Exponentially growing cultures of wild-type E. coli (strain 1), an *ftsH* V86F mutant (strain 8), an *lpxC* H19T mutant (strain 15), and an *ftsH*^V86F^*lpxC*^H19T^ double mutant (strain 16) were treated with 1× MIC ampicillin in the presence/absence of 1/3× MIC lysine hydrochloride for the indicated times, after which samples were taken to determine CFU and percent survival. Data represent the mean from 3 biological replicates; error bars indicate standard deviations.

The main function of FtsH is to degrade LpxC, which is involved in the first, irreversible step of lipid synthesis that regulates the synthesis of lipopolysaccharide (LPS) (27–29). When we quantified LPS levels using silver staining SDS-PAGE to indirectly detect LpxC enzyme activity changes due to the *ftsH* V86F mutation, we found that the LPS content in the *ftsH* V86F mutant was about half that found in the wild-type strain (Fig. 5a). The wild-type level of LPS was largely restored by replacing the *ftsH* mutant allele with the homologous wild-type gene (Fig. 5a). These data indicate that the *ftsH* V86F allele encodes a gain-of-function enzyme that causes greater degradation of LpxC.

To determine whether the reduced LPS levels account for tolerance to the lysine hydrochloride-ampicillin combination, we overexpressed *lpxC* to increase the synthesis of LPS by transferring the pBAD18-*lpxC* plasmid into the *ftsH* V86F mutant. Tolerance of the *ftsH* V86F mutant was largely reversed by overexpression of *lpxC* (Fig. 5b). LPS synthesis was also impeded by constructing an H19T allele of *lpxC*, which is known to reduce LPS production (30–32), in wild-type cells and in an *ftsH* V86F mutant. Both *lpxC* H19T and *lpxC* H19T-*ftsH* V86F mutants reduced lysine hydrochloride-stimulated ampicillin killing relative to that in wild-type cells, with the double mutant exhibiting slightly more tolerance to killing (Fig. 5c). The MIC of lysine hydrochloride also exhibited a gradual decrease with wild-type > *ftsH* V86F > *lpxC* H19T > *lpxC* H19T- *ftsH* V86F (Table S1): as the LPS content of the cell surface decreased, the bacteriostatic effect of lysine hydrochloride increased. We hypothesize that the *ftsH* V86F substitution reduces the lipopolysaccharide content in the outer membrane, which results in a reduction of a lysine hydrochloride-membrane interaction that in wild-type cells enhances the lethal action of β-lactams. Such a conclusion was further strengthened when adding exogenous LPS into the lysine hydrochloride-ampicillin combination treatment reduced enhancement of ampicillin killing by lysine hydrochloride (Fig. S9).

### ROS changes associated with enhancement of ampicillin lethality by lysine hydrochloride.

**FIG 6:**
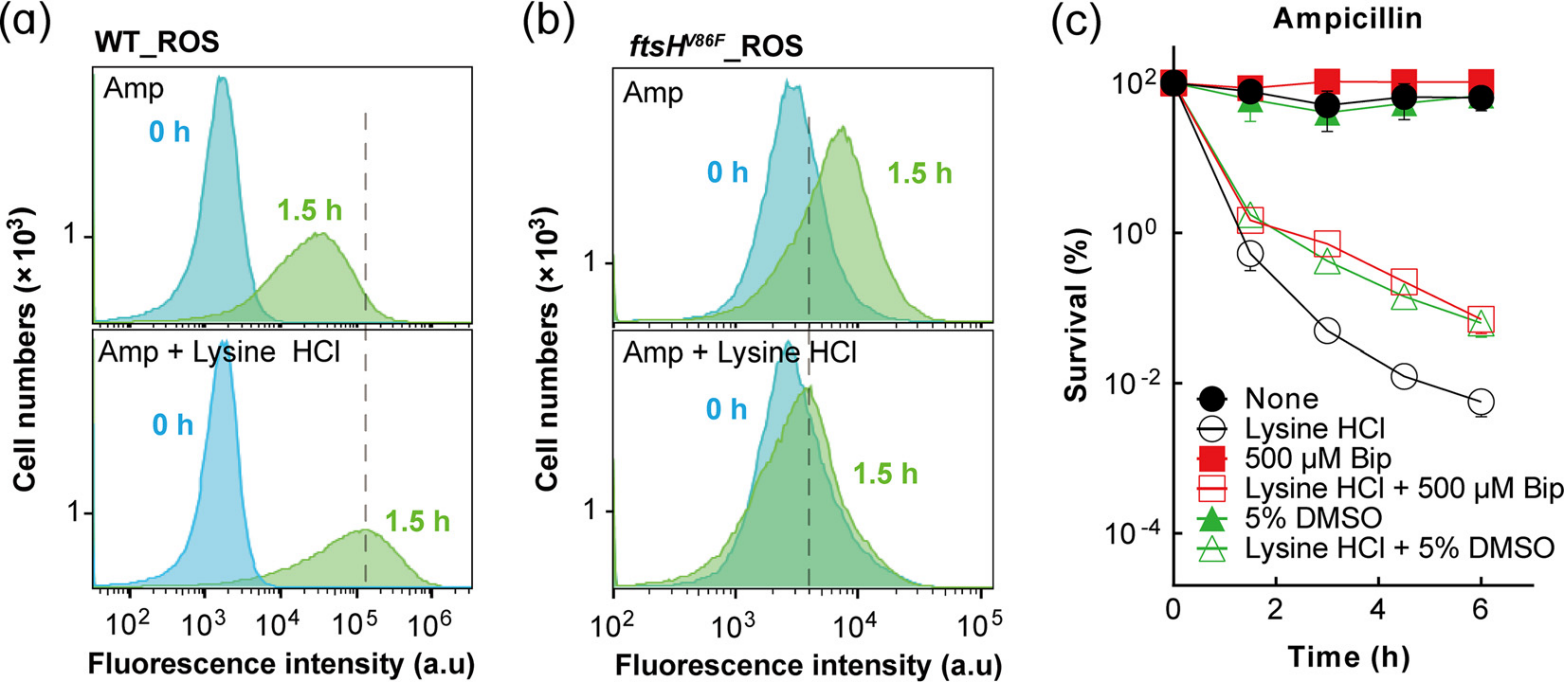
Association of intracellular ROS levels with lysine hydrochloride enhancement of ampicillin lethality. (a and b) Measurement of intracellular ROS accumulation during lysine-ampicillin combination treatment. Exponentially growing cultures of wild-type E. coli (strain 1) (a) and the *ftsH* V86F mutant (strain 8) (b) were pretreated with 5 μM carboxy-H_2_DCFDA for 20 min and then exposed to 1× MIC ampicillin (top) or 1× MIC ampicillin combined with 1/3× MIC lysine hydrochloride (bottom) for 1.5 h. Samples were then subjected for flow cytometry to assess fluorescence as an indicator of intracellular ROS level. Curves farther to the right indicate higher levels of ROS. Similar data were obtained from 3 biological replicates. (c) Chemical suppression of intracellular ROS accumulation and lysine hydrochloride enhancement of β-lactam lethality. Exponentially growing cultures of wild-type E. coli (strain 1) were pretreated with 5% DMSO or 500 μM bipyridyl (Bip) for 20 min before addition of ampicillin (1× MIC) or ampicillin plus lysine hydrochloride for the indicated times when samples were taken for CFU and percent survival determination. DMSO data are superimposed with wild-type data. Data represent the means from 3 biological replicates; error bars indicate standard deviations.

ROS have been identified as execution factors that contribute to killing by a variety of lethal insults (33–39). To explore whether ROS contribute to lysine-ampicillin combination-mediated killing, we measured ROS accumulation using the fluorescent dye carboxy-2′,7′-dichlorodihydrofluorescein diacetate (H_2_DCFDA) followed by flow cytometry. ROS levels in the lysine hydrochloride-containing combination group were elevated compared to those in the ampicillin-only group using wild-type cells (Fig. 6a) despite lysine hydrochloride treatment alone slightly reducing ROS (Fig. S10). Lower ROS levels were observed in the tolerant *ftsH* V86F mutant, in both the absence and the presence of lysine hydrochloride (Fig. 6b). Lysine hydrochloride cotreatment of the *ftsH* V86F mutant decreased (Fig. 6b) rather than increased (Fig. 6a) ROS as seen in wild-type cells. When ROS accumulation was lowered by adding ROS scavengers, such as dimethyl sulfoxide (DMSO) or the iron chelator 2,2′-bipyridyl, a large (e.g.,10- to 10,000-fold) reduction in lysine hydrochloride-mediated enhancement of β-lactam killing was observed (Fig. 6c and Fig. S11).

As reported previously, stress-mediated accumulation of ROS in E. coli depends largely on electron transfer chain activation during respiration (38). Therefore, production of ROS can also be assessed indirectly by determining the expression changes of relevant genes in the tricarboxylic acid (TCA) cycle. Comparison of ampicillin treatment with the lysine hydrochloride-ampicillin combination using wild-type cells revealed significantly increased expression of several TCA cycle genes for the combination treatment (Fig. S12a), while lysine hydrochloride treatment alone did not cause such increases (Fig. S12b). Such elevated gene expression was absent or was even reversed with the tolerant *ftsH* V86F mutant undergoing the same treatment (Fig. S12c). These findings, which are consistent with fluorescence dye detection of ROS (Fig. 6a and b), suggest that elevated TCA cycle activity contributes to increased ROS accumulation and cell death. Such a conclusion was further supported when malonate, an inhibitor of a TCA cycle enzyme (17), or a deficiency in *acnB*, a gene encoding a key enzyme of the TCA cycle, eliminated lysine hydrochloride stimulation of ampicillin killing (Fig. S13).

### Multiple Gram-negative bacteria show lysine hydrochloride-enhanced killing by β-lactams.

To determine whether lethality enhancement by lysine hydrochloride is generally applicable, pathogenic E. coli (Sakai), Acinetobacter baumannii ATCC 17978, and Pseudomonas aeruginosa (UCBPP-PA14) were examined for MIC and FICI values. Lysine hydrochloride showed additive effects for β-lactams (ampicillin, meropenem, and ceftriaxone) with all three bacteria (Table 3). β-Lactams combined with lysine hydrochloride showed an indifferent effect with K. pneumoniae (Kpn43816), which may be due to a thick bacterial capsule blocking lysine hydrochloride interaction with the outer membrane (Table 3). Staphylococcus aureus RN450, a Gram-positive bacterium that lacks LPS, also showed an indifferent effect for ampicillin, ciprofloxacin, and kanamycin when combined with lysine hydrochloride (Table 3). The last two bacterial species were not studied further.

**TABLE 3:** Effect of lysine hydrochloride on β-lactam susceptibility of several Gram-negative bacteria and S. aureus

Strain	β-Lactam tested	MIC^*e*^		FICI	Interpretation
		Alone	Combination^*a*^		
E. coli Sakai^*b*^	Ampicillin	1.5	0.75	1	Additive
	Meropenem	0.04	0.01	0.75	Additive
	Ceftriaxone	0.03	0.015	1	Additive
A. baumannii ATCC 17978^*b*^	Ampicillin	16	2	0.625	Additive
	Meropenem	0.4	0.05	0.625	Additive
	Ceftriaxone	24	3	0.625	Additive
P. aeruginosa PA14^*b*^	Ampicillin	80^*c*^	10	0.625	Additive
	Meropenem	0.4	0.1	0.75	Additive
	Ceftriaxone	8	2	0.75	Additive
K. pneumoniae Kpn43816	Ampicillin	16	16	1.125	Indifferent
	Lysine HCl	1	0.125		
	Meropenem	0.04	0.04	2	Indifferent
	Lysine HCl	1	1		
	Ceftriaxone	0.04	0.04	1.25	Indifferent
	Lysine HCl	1	0.25		
S. aureus RN450^*d*^	Ampicillin	0.75	0.75	1.5	Indifferent
	Lysine HCl	1.5	0.75		
	Kanamycin	7.5	7.5	1.125	Indifferent
	Lysine HCl	1.5	0.188		
	Ciprofloxacin	0.125	0.125	1.125	Indifferent
	Lysine HCl	1.5	0.188		

a MIC when the antibiotic was tested in combination with 0.5 M lysine hydrochloride (lysine HCl) for E. coli Sakai, A. baumannii ATCC 17978, and P. aeruginosa PA14; for K. pneumoniae Kpn43816 and S. aureus RN450, combination MIC for each antimicrobial was determined in the presence of lysine HCl at the concentration listed immediately below that value.

b Lysine hydrochloride MIC was 1 M for these bacterial species.

c MIC determined in LB broth was lower than that (300 μg/mL) determined in Mueller-Hinton broth.

d Strain RN450 was chosen due to its susceptibility to β-lactams. Most other S. aureus strains are nonsusceptible to β-lactams.

e MICs are in micrograms per milliliter except for those of lysine hydrochloride, which are molar concentrations.

**FIG 7:**
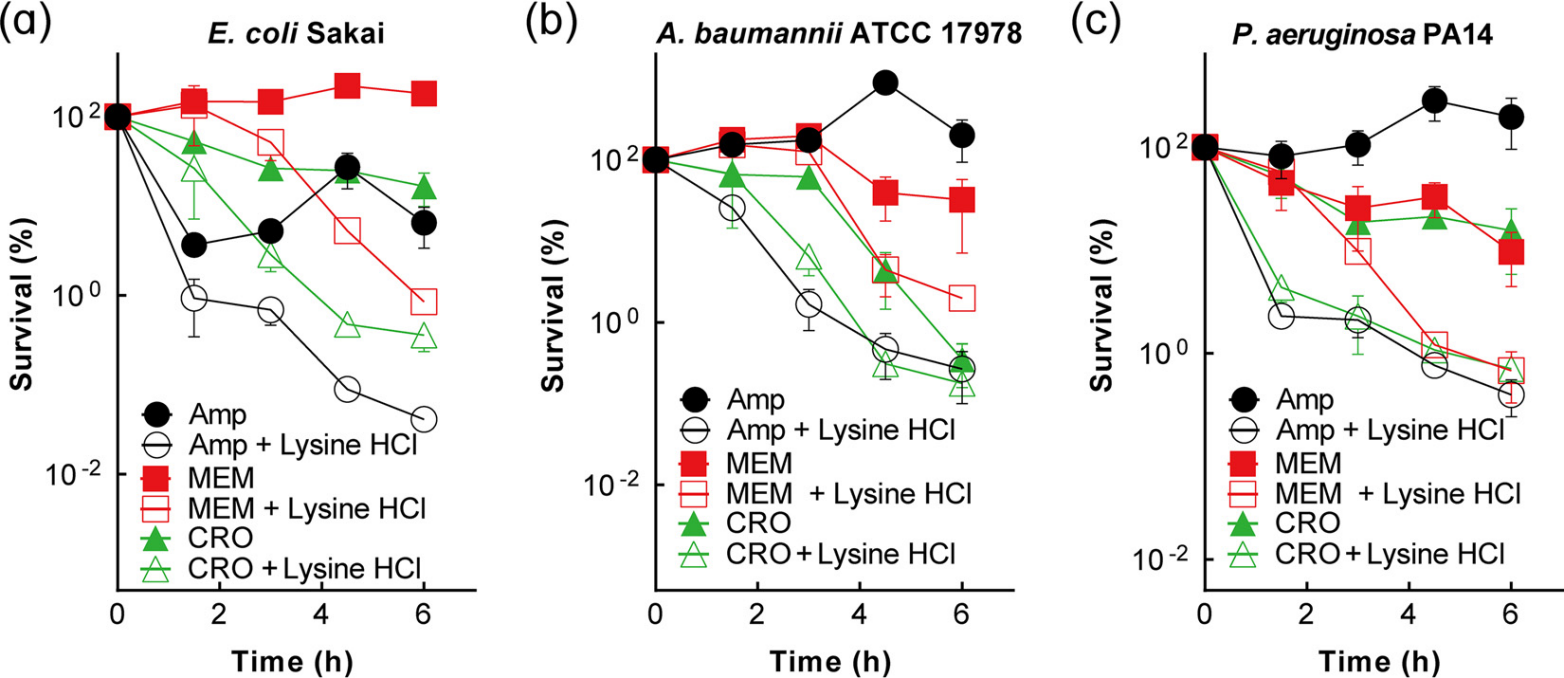
Enhancement of β-lactam killing by lysine hydrochloride with pathogenic Gram-negative bacteria. (a to c) Enhancement of β-lactam lethality by lysine hydrochloride with pathogenic E. coli Sakai (a), Acinetobacter baumannii ATCC 17978 (b), and Pseudomonas aeruginosa UCBPP-PA14 (c). Exponentially growing bacterial cultures were treated with β-lactams (1× MIC ampicillin [Amp], 2× MIC meropenem [MEM], or 2× MIC ceftriaxone [CRO]) alone or in combination with 0.33 M (1/3× MIC) lysine hydrochloride for the indicated times, after which samples were taken for determination of CFU and percent survival. Data represent the means from 3 biological replicates; error bars indicate standard deviations.

When a kinetic killing assay for the lysine hydrochloride–β-lactam combination was performed, E. coli Sakai, A. baumannii ATCC 17978, and P. aeruginosa PA14 displayed enhanced killing by the combination (Fig. 7). At the lysine concentration used, there was little effect on growth of the 3 bacterial species (Fig. S14). Thus, the enhancement of β-lactam lethality by lysine hydrochloride is applicable to many Gram-negative bacterial species.

### Arginine enhances β-lactam-mediated killing.

**FIG 8:**
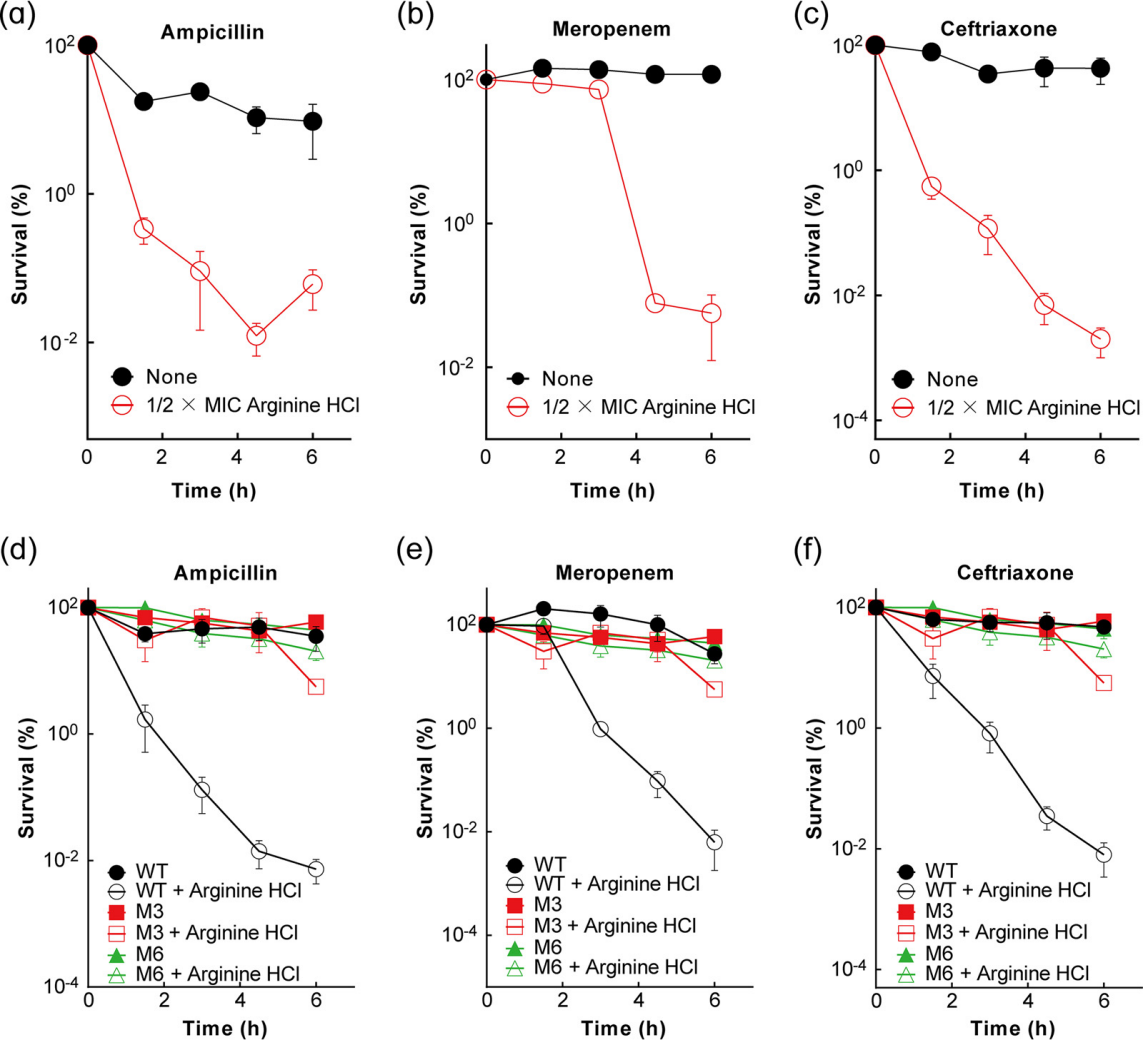
Enhancement of β-lactam lethality by arginine hydrochloride. (a to c) Effect of subinhibitory concentrations of arginine hydrochloride on lethal action of several β-lactams. Exponentially growing cultures of wild-type E. coli BW25113 were treated with 1× MIC ampicillin (a), 2× MIC meropenem (b), or 2× MIC ceftriaxone (c) in the presence or absence of 1/2× MIC arginine hydrochloride for the indicated times. Percent survival was determined by CFU measurement of samples taken at the time of drug addition. (d to f) Elimination of arginine hydrochloride-mediated enhancement of β-lactam killing by tolerant mutant. Experimental conditions were as for panels a to c, but two tolerant mutants, M3 (strain 18) and M6 (strain 19), enriched from arginine-ampicillin combination treatment, were compared with wild-type cultures (strain 1) for arginine hydrochloride–β-lactam killing. Data represent the means from 3 biological replicates; error bars indicate standard deviations.

We also examined two other basic amino acids, arginine and histidine, for enhancement of β-lactam activity. When the neutralized forms (hydrochloride salt) of the two compounds were tested, arginine hydrochloride (Fig. 8), but not histidine hydrochloride (Fig. S15), enhanced β-lactam killing similar to that seen with lysine hydrochloride (Fig. 1). A combination of 1/2× MIC arginine hydrochloride with β-lactams (ampicillin, meropenem, or ceftriaxone) reduced survival 100- to 10,000-fold over the β-lactam-alone control (Fig. 8a to c). No growth inhibition by arginine hydrochloride alone at concentrations at or below 1/2× MIC was observed (Fig. S16).

Arginine hydrochloride enhanced β-lactam-mediated killing in a way that closely mimicked lysine hydrochloride effects. For example, uptake of arginine hydrochloride was not needed for lethality enhancement (Fig. S17a), and stabilization of outer membrane by exogenous cations, CaCl_2_ and MgCl_2_, reversed arginine hydrochloride-mediated lethality enhancement (Fig. S17b and c). Moreover, tolerant mutants selected against arginine hydrochloride-ampicillin killing harbored mutations mapping in *ftsH*, the gene responsible for lysine hydrochloride–β-lactam tolerance (Table 2 and Table S3), although the allele was different (V46A or V41G [Table S3]). These tolerant mutants not only reversed arginine hydrochloride-mediated killing enhancement (Fig. 8d to f) but also eliminated the enhancing effect of lysine hydrochloride (Fig. S18d to f). Likewise, the *ftsH* V86F mutant, which was enriched for lysine hydrochloride-ampicillin tolerance, reduced killing by the arginine hydrochloride–β-lactam combination (Fig. S18a to c). Thus, hydrochlorides of arginine and lysine stimulate β-lactam lethality in similar ways.

## DISCUSSION

**FIG 9:**
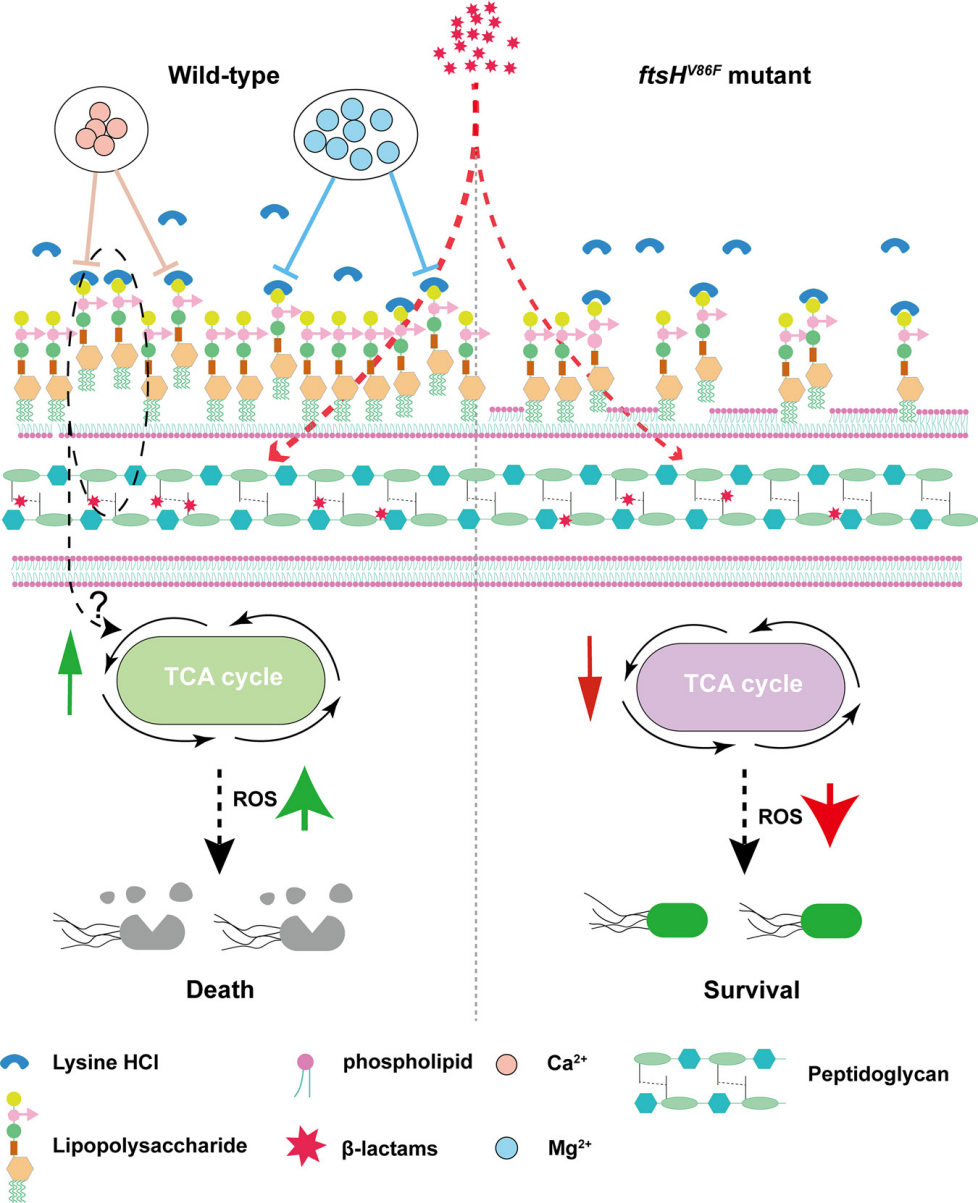
Schematic description of enhancement of β-lactam lethality by lysine hydrochloride. Lysine hydrochloride destabilizes the outer membrane by binding to lipopolysaccharide (LPS). This membrane stress stimulates the TCA cycle by a mechanism that needs further investigation but is possibly similar to that reported with polymyxin (55, 56), thereby raising intracellular ROS levels. Increased ROS, plus slightly increased β-lactam accessibility to the site of peptidoglycan synthesis due to outer membrane damage, elevate β-lactam lethality. Divalent ions, such as Ca^2+^ and Mg^2+^, inhibit lysine hydrochloride-enhanced β-lactam lethality by lowering lysine hydrochloride binding to LPS. An *ftsH* mutation reverses lysine hydrochloride-enhanced β-lactam lethality by reducing LPS content, thereby reducing the level of lysine hydrochloride bound to the outer membrane.

The present work demonstrated enhancement of β-lactam activity by lysine hydrochloride through interaction with LPS and destabilization of the outer membrane. As illustrated in Fig. 9, these events may stimulate β-lactam-mediated killing in two ways. In one, a destabilized outer membrane allows β-lactams to more readily access the cell wall and inhibit new wall synthesis. That would exacerbate the fragility of the wall weakened by β-lactam action. In another scenario, outer membrane destabilization by lysine hydrochloride would trigger an increase in intracellular ROS accumulation upon ampicillin exposure, thereby adding to ROS induced by β-lactam treatment alone (36, 38). Elevated ROS levels, which derive from stimulation of the TCA cycle and oxidative phosphorylation (38), enhance β-lactam killing.

Enhancement was observed at both the bacteriostatic level (growth inhibition by lysine hydrochloride and by ampicillin was additive) and the bactericidal level (noninhibitory concentrations of lysine hydrochloride enhanced ampicillin-mediated killing at concentrations at or above the normalized MIC). The small increase of bacteriostatic effect likely derives from membrane perturbation, while enhancement of killing derives additionally from ROS effects. The action of lysine hydrochloride and β-lactam can be viewed as independent, but when they are combined, they cause enough membrane and wall damage to stimulate an additive/synergistic ROS-mediated stress response.

Examination of a tolerant mutant (reduced killing, no change in MIC) identified the FtsH/HflkC membrane protein degradation complex as a participant in lysine-enhanced lethality of β-lactams. The mutant gene, *ftsH*, regulates LPS synthesis by degrading LpxC, a critical enzyme for that synthesis. The decrease of LPS content in the outer membrane of the tolerant mutant implies enhanced *ftsH* V86F-mediated degradation activity, indicating that the mutation is a gain-of-function mutation. This notion is supported by failure of the plasmid-borne wild-type allele to complement the mutant phenotype and by tolerance being observed when the mutant copy was expressed from a plasmid in a wild-type background (Fig. S19). Thus, tolerance can derive from decreasing the LPS content of the outer membrane. As expected, increasing the synthesis of LPS by overexpressing *lpxC* in the tolerant mutant substantially lowered protection due to the *ftsH* V86F substitution; further reduction of LPS synthesis by a mutation in *lpxC* increased the tolerance of the mutant.

Lysine hydrochloride may act as a monovalent cation that binds to negatively charged groups on LPS and thereby perturbs the outer membrane. The level of lysine-LPS interaction, not the level of LPS or lysine hydrochloride alone, is responsible for membrane perturbation and the killing enhancement phenotype, because divalent ions, such as Ca^2+^ and Mg^2+^, reversed the stimulating effect of lysine on β-lactam lethality. These ions likely stabilize the outer membrane by competing with lysine for LPS binding; a mutation (V86F) in *ftsH*, which leads to reduced levels of outer membrane LPS available for lysine to interact with, would reverse and indeed did reverse lysine HCl enhancement of ampicillin killing.

Interaction of lysine HCl with LPS enhanced the TCA cycle and ROS production in the presence but not the absence of ampicillin (Fig. 6a and Fig. S10a and S12a and b), which would account for enhanced β-lactam lethality with wild-type cells. An *ftsH* (V86F) mutation, which resulted in reduction of LPS content through enhanced degradation of LpxC, reversed both the increased expression of TCA cycle-related genes (Fig. S12c) and elevated intracellular ROS accumulation (Fig. 6b). That protected the *ftsH* (V86F) mutant from enhanced killing by the lysine hydrochloride-ampicillin combination. The fact that lysine HCl enhances the lethality of wall-damaging β-lactam antibiotics, but not that of antimicrobials (e.g., fluoroquinolones and aminoglycosides) that do not damage cell wall, suggests a synergistic/additive effect of lysine HCl-triggered outer membrane destabilization and β-lactam-mediated wall damage (Fig. S10). Overall, the lysine–β-lactam system represents a new membrane-based way to exacerbate β-lactam-elicited wall damage and to stimulate ROS-mediated bacterial self-destruction characteristic of many lethal stressors (38–40). Involvement of ROS is supported by ROS scavengers and iron chelators, added to cultures before drug treatment, improving bacterial survival. A precedent for stimulation of ROS accumulation by membrane damage has been observed with insertion of the MalE-LacZ fusion protein into E. coli membranes (41) and with polymyxin B treatment (42). The absence of complete mitigation of ROS-based effects indicates that direct cellular damage, such as cell lysis, also contributes to the death process.

Enhancement of the TCA cycle can supply NADH that fuels the oxidative respiration that generates ROS as by-products. Indeed, increased expression of genes involved in the TCA cycle and oxidative phosphorylation has been reported upon treatment with many lethal stressors (35, 36, 38, 39); the present work also found that expression of several TCA cycle genes (*fumA*, *fumB*, *sucB*, and *sucD*) was elevated by lysine hydrochloride in the wild-type cells but not in the lysine-ampicillin-tolerant mutant when bacterial cells were treated with ampicillin. Involvement of elevated TCA in lysine HCl-enhanced ampicillin killing is also supported by both malonate, an inhibitor of TCA cycle (17), and a deficiency in *acnB* (encoding a key enzyme of the TCA cycle) reducing/eliminating enhancement of ampicillin killing. These data support the idea that lysine enhancement of β-lactam killing derives mainly from elevated central metabolism and the subsequent accumulation of intracellular ROS.

We found that lethality enhancement by lysine hydrochloride is applicable to several Gram-negative bacteria, with the notable exception of K. pneumoniae. With this bacterium the absence of activity may derive from the extracellular capsule blocking lysine binding to LPS. As expected, lysine had little effect with S. aureus, which lacks LPS. We also found that a similar β-lactam lethality-stimulating effect can be expanded to arginine but not histidine. These data help expand the scope of our work to multiple bacterial species and 2 of the 3 basic amino acids.

We note that the serum concentration of lysine in adult humans is 164 to ~192 μM (43–45), which is below the concentration of lysine hydrochloride used in the present work. It may be possible to artificially raise that lysine concentration during β-lactam treatment. One approach is to fix lysine hydrochloride to the surface of nanomaterials to increase the local concentration and binding efficiency, thereby bypassing the high concentration requirement for lysine-mediated lethality enhancement. Since lysine hydrochloride shows no additive or synergistic effect with aminoglycosides, a previous report of enhanced aminoglycoside lethality due to l-lysine (19) likely reflects the alkaline property of lysine. Many amino-rich compounds, such as polyamines, have shown antimicrobial activity (46–51), but the mechanisms underlying such antimicrobial effects are still obscure. The present work can guide refinement of such compounds, since a similar mode of action may underly their antimicrobial effects.

## MATERIALS AND METHODS

### Bacterial strains and reagents.

Isogenic E. coli K-12 strains (see Table S1 in the supplemental material) were obtained from the Keio collection (52) or constructed using CRISPR genome editing technology (53). Plasmids and primers used for this work are listed in Table S4. E. coli strains were cultured by shaking at 200 rpm aerobically at 37°C in Luria-Bertani (LB) broth or by plating on LB agar incubated at 37°C. Yeast extract, tryptone, agar, and 5(6)-carboxy-2′,7′-dichlorodihydrofluorescein diacetate (carboxy-H_2_DCFDA) were obtained from Thermo Fisher Scientific Corp. (Waltham, MA, USA). Ciprofloxacin, ampicillin, and kanamycin were obtained from Sigma-Aldrich Corp. (St. Louis, MO, USA). Vancomycin, erythromycin, lysine monohydrochloride, arginine hydrochloride, histidine hydrochloride, malonate, propidium iodide, proteinase K solution, 5× protein loading dye, dimethyl sulfoxide (DMSO), and 2.2′-bipyridyl were purchased from Sangon Biotech Inc. (Shanghai, China). Lipopolysaccharide (LPS) was bought from Beyotime Biotech Inc. (Jiangsu, China). Ceftriaxone (Roche, Shanghai, China), and meropenem (Shenghuaxi Pharmaceutical Co., Chongqing, China) were gifts from the Zhongshan Hospital (Xiamen, China) pharmacy.

### Susceptibility determination.

Overnight cultures were diluted 1:100 into fresh LB/Mueller-Hinton (MH; for MIC determination with Pseudomonas aeruginosa) medium and grown to early exponential phase (optical density at 600 nm [OD_600_], 0.25 to 0.3). They were further diluted 1:5,000 in fresh LB broth to about 10^5^ cells/mL. Measurement of MIC used a staggered 2-fold broth dilution method according to CLSI protocol (54). After incubation for 24 h, MIC was determined as the lowest concentration that inhibited culture growth judged by visible turbidity (Table S2).

The fractional inhibitory concentration index (FICI) procedure was as follows. Cultures were mixed with two test compounds at various concentrations according to a checkerboard assay using 96-deep-well culture plates. Inoculated plates were incubated at 37°C for 24 h. FICI was determined as the lowest concentration combination that showed no visible turbidity. (FICI ≤ 0.5 is defined as synergy, 0.5 < FICI ≤ 1 as additive, 1 < FICI < 4 as indifference, and FICI ≥ 4 as antagonism).

### Bacterial killing assays.

Overnight cultures of E. coli were diluted 1:100 into fresh LB liquid medium and grown to early exponential phase (OD_600_, ~0.25 to 0.3) at 37°C, followed by aliquoting into glass tubes and treatment with various concentrations (normalized to MIC) of β-lactams or β-lactam plus lysine hydrochloride, arginine hydrochloride, or histidine hydrochloride for the indicated times. Aliquots were serially diluted, and 10-μL aliquots of each dilution were plated on drug-free agar for enumeration of CFU after incubation of agar plates for 24 h at 37°C. Percent survival was determined relative to CFU of a sample taken at the time of drug addition. DMSO, bipyridyl, CaCl_2_, MgCl_2_, and malonate were added at non- or subinhibitory concentrations 20 min before treatment with antibiotics or antibiotic-lysine hydrochloride combinations. LPS at 0.66 mg/mL was incubated with or without lysine hydrochloride at 37°C for 30 min before the ampicillin addition to E. coli BW25113 cultures.

### FESEM.

E. coli overnight cultures were diluted 1:100 in fresh LB liquid medium and grown to an OD_600_ of ~0.25 to 0.3. Samples were left untreated or treated with lysine hydrochloride (0.33 M) before cells were harvested by centrifugation at 8,000 × *g* for 5 min, followed by washing of the cell pellet twice with LB medium. Cell pellets were fixed by suspension in 5% formaldehyde plus 2% glutaraldehyde; they were then incubated overnight at 4°C. The supernatant of the fixed samples was discarded after centrifugation at 2,000 × *g* for 10 min. The precipitate was washed three times with 0.1 M phosphate buffer (pH 7.2) and then resuspended at 10^9^ cells/mL in 0.1 M phosphate buffer (pH 7.2). The suspension was quickly applied and attached to the adhesive on glass slides and dried for several minutes. Samples on the dried glass slides were processed on ice for dehydration by placing slides sequentially in 30% ethanol for 5 min, 50% ethanol for 5 min, 70% ethanol for 10 min, 80% ethanol for 10 min, 95% ethanol for 15 min, and 100% ethanol (pretreated with anhydrous sodium sulfate) for 15 min. Samples were then sprayed with gold and finally examined by field emission scanning electron microscopy (FESEM; SUPRA55 SAPPHIRE microscope; Zeiss, Germany).

### Enrichment of tolerant mutants.

Overnight cultures of E. coli were diluted 1:100 in 5 mL of LB medium and incubated for 1.5 h at 37°C. Then a sample was removed for CFU determination, and the remainder of the culture was treated with a combination of 0.33 M lysine hydrochloride and 1× MIC ampicillin for 4.5 h. This treatment resulted in a 100-fold reduction in survival. A sample was taken, serially diluted, and plated on agar for CFU determination. The remaining cultures were centrifuged at 8,000 × g for 5 min to concentrate the cells. Cells were washed twice with LB medium to remove drug; they were then resuspended in fresh 5 mL of LB broth and cultured to mid-exponential phase at 37°C for another round of treatment with the lysine hydrochloride-ampicillin combination. After 10 rounds of enrichment, bacterial survival increased approximately 100-fold compared with that of wild-type cells. These cultures were washed, diluted, and plated onto drug-free agar to obtain single colonies for further screening to identify tolerant mutants. Cultures grown from the single colonies were subjected to both MIC determination and killing by ampicillin alone and by ampicillin-lysine hydrochloride combinations. Mutants having an MIC equal to that of the parental strain and reduced enhancement of ampicillin lethality by lysine hydrochloride were candidates for further analysis.

### Whole-genome sequencing.

Two confirmed tolerant mutants, LYS1 and LYS3, were incubated in 5 mL of LB medium at 37°C overnight. Genomic DNA was extracted from overnight cultures using a bacterial chromosome DNA isolation kit (Tiangen Biotech Co., Beijing, China). DNA samples were sent to Majorbio Co., Ltd. (Shanghai, China), for whole-genome resequencing and comparative sequence analysis against the E. coli BW25113 whole-genome sequence (GenBank accession number CP009273.1).

### Mutant construction.

Mutations identified by whole-genome sequencing were reconstructed by CRISPR-based allelic exchange in the parental strain (53).

### Measurement of fluorescence by flow cytometry.

Intracellular bacterial fluorescence intensity was determined using fluorescence-based flow cytometry with a CytoFLEX A00-1-1102 flow cytometer (Beckman Coulter Inc.; distributed by Suzhou Xitogen Biotechnologies Co., Ltd., Suzhou, China). Exponentially growing cultures of wild-type and *ftsH* V86F mutant cells were treated with carboxy-H_2_DCFDA (10 μM final concentration) or propidium iodide (1-μg/mL final concentration) for 20 min before addition of ampicillin, lysine hydrochloride, lysine hydrochloride-ampicillin combination, ciprofloxacin, or lysine hydrochloride-ciprofloxacin combination. Samples (500 μL), taken at the indicated times, were washed with prechilled saline using centrifugation (8,000 × *g*, 4°C, 3 min), chilled on ice, and examined by flow cytometry. A total of 200,000 cells were analyzed at a 30-μL/min rate for each sample to measure fluorescence. Detection parameters were 20 mV of laser power and a 525/40-nm band-pass filter (fluorescein isothiocyanate [FITC] channel) or 582/42-nm band-pass filter (phycoerythrin [PE] channel). Data were analyzed using FlowJo software. All flow cytometry data were obtained with 3 biological replicates; one representative data set is presented in the main text figures and the others in figures in the supplemental material.

### Quantification of LPS.

Exponentially growing cultures of the wild type, *ftsH* V86F mutant, and *ftsH* V86F mutant complemented with the homologous wild-type *ftsH* allele were harvested and washed by repeated centrifugation and resuspension in phosphate-buffered saline (PBS). Cell lysates were prepared by sonication (sonicate for 10 s every 10 s, with a total duration of 1 min) and centrifugation (10,000 × *g*, 10 min). Protein concentrations were measured using an enhanced bicinchoninic acid (BCA) protein assay kit (Beyotime Biotech Inc., Shanghai, China). Twenty-five micrograms of total protein of samples was mixed with 5× protein loading dye and boiled for 10 min. Proteinase K solution (2.5-mg/mL final concentration) was added to the samples, followed by incubation at 60°C for 1 h and then centrifugation (16,000 × *g*, 30 min). The supernatant fluids were loaded onto 15% tricine-SDS polyacrylamide gels, and the samples were resolved by electrophoresis at 150 V for 1 h. LPS levels were measured using a fast silver stain kit (Beyotime Biotech Inc., Shanghai, China) according to the vendor’s technical manual. LPS was quantified using ImageJ densitometric software.

### Measurement of gene expression by RT-PCR.

Overnight cultures of the wild type and the *ftsH* V86F mutant were diluted 100-fold in 30 mL of LB liquid medium at 37°C and incubated with rotary shaking at 200 rpm to mid-log phase. Cultures were then treated with 1× MIC ampicillin, lysine hydrochloride, or a combination of 1/3× MIC lysine hydrochloride and 1× MIC ampicillin for 0 or 60 min, followed by centrifugation (4,600 × *g* for 10 min at 4°C) to recover bacterial cells. Total RNA was extracted using an RNA extraction kit (TransGen Biotech Co., Beijing, China) and reverse transcribed using random primers, and single-stranded cDNAs were synthesized according to protocols of the RNA reverse transcription kit (Applied Biological Materials, Richmond, Canada). The synthesized cDNA was diluted to 100 ng/mL, followed by real-time PCR (RT-PCR) using primers listed in Table S4 and the following thermal cycling protocol: 1 cycle at 95°C for 30 s and then 40 cycles of 95°C for 5 s followed by 60°C for 30 s. Primers for 16S rRNA were used as an internal reference for quantitative fluorescence RT-PCR determination of the relative transcription level for genes of interest.

### Statistical analyses.

At least three biological replicates were obtained for every experiment. Each data point in figures represents the mean of independent replicate experiments; error bars represent standard deviations of the means. A two-tail paired Student *t* test was used for comparison of TCA cycle gene expression in the wild-type strain or in *ftsH* V86F mutant under different treatment conditions. Significant differences were concluded when *P* values of <0.05 were achieved.

### Data availability.

Raw sequence data have been deposited to the Sequence Read Archive with BioProject number PRJNA974919.
