# Supplementary material for: Enhancement of β-Lactam-Mediated Killing of Gram-Negative Bacteria by Lysine Hydrochloride
Source: Microbiol Spectr. 2023 Jun 13;11(4):e01198-23. doi: 10.1128/spectrum.01198-23 (PMC10434284; doi:10.1128/spectrum.01198-23)
Supplement: Supplemental file 1 — Supplemental material. Download spectrum.01198-23-s0001.pdf, PDF file, 1.9 MB [file spectrum.01198-23-s0001.pdf]

# Supplementary Materials

## Enhancement of β-lactam-mediated killing of Gram-negative bacteria by lysine hydrochloride

Shouqiang Hong[a], Shaopeng Su[a], Qiong Gao[b], Miaomiao Chen[a], Lisheng Xiao[a], Runbo Cui[a], Yinli Guo[a], Yunxin Xue[a], Dai Wang[a], Jianjun Niu[c*], Haihui Huang[b*], and Xilin Zhao[a*]

[a]State Key Laboratory of Molecular Vaccinology and Molecular Diagnostics, Department of Laboratory Medicine, School of Public Health, Xiamen University, Xiamen, Fujian Province, China

[b]Institute of Antibiotics, Huashan Hospital, Fudan University, 12 Middle Wulumuqi Road, Shanghai 200040, China.

[c]Center of Clinical Laboratory, Zhongshan Hospital, School of Medicine, Xiamen University, 209 South Hubin Road, Siming District, Xiamen, Fujian Province, 361004, China.

## Table of Contents

**Supplementary Figures**

**Fig. S1**. Lysine lethality against *E. coli* abolished by neutralization.

**Fig. S2**. Effect of lysine HCl concentration on bacterial growth and enhancement of β-lactam-mediated killing.

**Fig. S3**. Lack of lethality enhancement of β-lactam-mediated killing by low concentrations of lysine HCl.

**Fig. S4**. Lysine HCl exhibited little lethality enhancement with kanamycin or ciprofloxacin.

**Fig. S5**. Transporter deficiency does not affect lysine HCl enhancement of ampicillin lethality.

**Fig. S6**. Lysine HCl damages bacterial cell surface.

**Fig. S7**. Neither a deficiency nor overexpression of *gntK* affects lysine stimulation of β-lactam killing.

**Fig. S8**. The *ftsH* V86F mutation affects neither bacterial growth nor ampicillin lethality.

**Fig. S9**. Pre-incubation with LPS reduced lysine HCl enhancement of ampicillin lethality.

**Fig. S10**. Measurement of intracellular ROS accumulation during lysine HCl- ampicillin and lysine-ciprofloxacin combination treatment.

**Fig. S11**. Protection from a lysine HCl-ampicillin combination-mediated killing by chemical suppression of ROS.

**Fig. S12.** An *ftsH* V86F mutation eliminated the upregulation of several TCA cycle genes by the ampicillin-lysine HCl combination.

**Fig. S13.** Suppression of lysine HCl enhancement of β-lactam lethality by malonate addition and

by a deficiency in *acnB*.

**Fig. S14.** Little growth inhibition of pathogenic Gram-negative bacteria treated with the lysine HCl concentration used for lethality enhancement.

**Fig. S15**. Little Lethality enhancement of β-lactam-mediated killing by histidine HCl.

**Fig. S16**. Effect of arginine HCl concentration on *E. coli* growth.

**Fig. S17**. Arginine HCl enhances ampicillin-mediated killing by destabilizing outer membrane.

**Fig. S18**. Cross tolerance to enhancement of β-lactam lethality between mutants selected against lysine HCl- and arginine HCl-ampicillin combinations.

**Fig. S19.** An *ftsH* V86F mutant allele confers gain-of-function phenotype.

## Supplementary Tables

**Table S1**. Bacterial strains and MIC for ampicillin, lysine HCl, arginine HCl.

**Table S2**. Minimal Inhibitory Concentrations (MIC) of compounds tested

**Table S3**. Mutational information of drug combination-tolerant mutants

**Table S4.** Primers used in the study

## Supplementary References

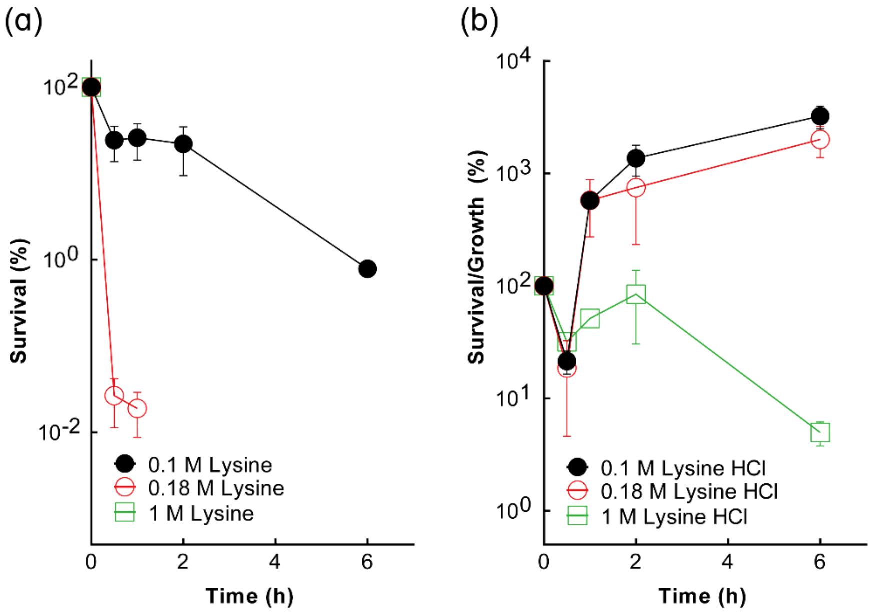


**Fig. S1. Lysine lethality against *E. coli* abolished by neutralization.** Exponentially growing cultures of wild type were treated with 0.1 M, 0.18 M, or 1 M lysine **(a)** or lysine HCl **(b),** respectively, for the indicated times after which samples were taken, serially diluted, and plated on drug-free agar for enumeration of bacterial colonies. Percent survival was determined using bacterial sample obtained at the time of lysine addition as control. CFU was below the detection limit when treated with 1 M lysine for 30 min. Data represent the mean from 3 biological replicates; error bars indicate standard deviations.

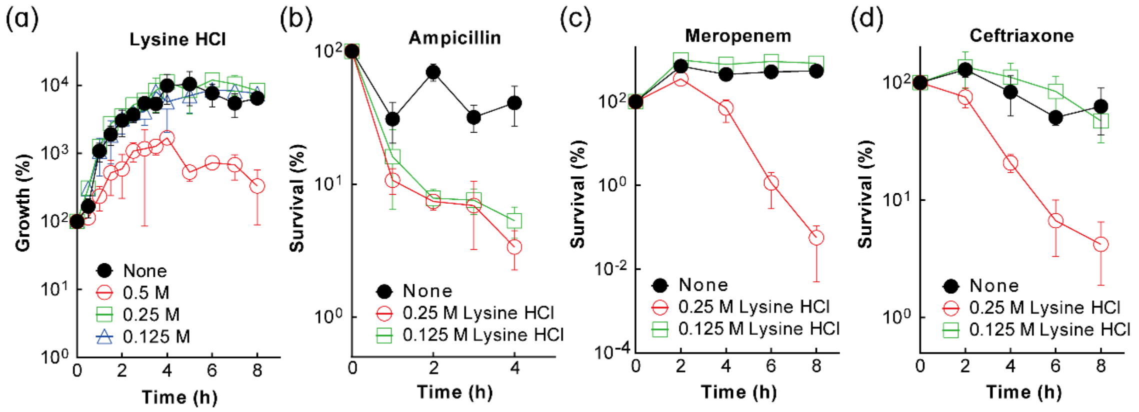


**Fig. S2. Effect of lysine HCl concentration on bacterial growth and enhancement of β-lactam-mediated killing. (a)** Effect of lysine concentration on bacterial growth. Overnight cultures of wild type *E. coli* cells were diluted 100-fold into fresh LB broth and incubated aerobically at 37°C for 1 h before lysine HCl was added at 1/8, 1/4, 1/2 M (MIC). Cells were further incubated for the indicated times before aliquots were taken for colony-forming unit determination. Percent survival (growth) was calculated using a sample taken at the time of lysine addition as control. **(b-d)** Effect of lysine concentration on enhancement of β-lactams mediated-killing. Exponentially growing wild-type cultures were treated with 1 MIC ampicillin (**b**), 2 MIC meropenem **(c)**, or 2 MIC ceftriaxone **(d)** in the presence of the indicated concentrations of lysine HCl for the indicated times after which samples were processed as in panel **a**. Data represent the mean from 3 biological replicates; error bars indicate standard deviations.

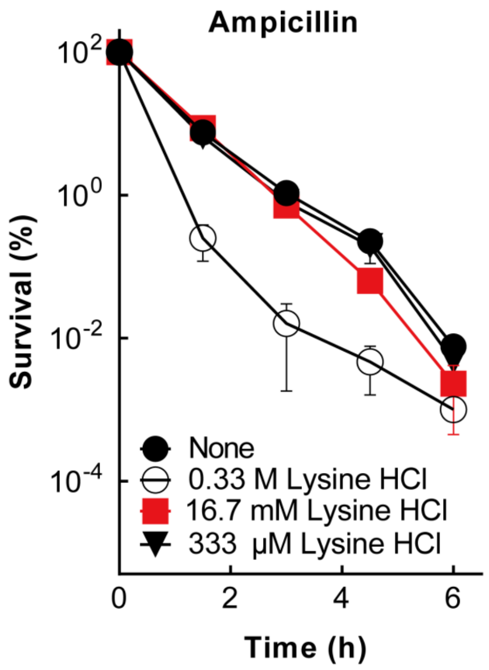


**Fig. S3. Lack of lethality enhancement of β-lactam-mediated killing by low concentrations of lysine HCl.** Exponentially growing cultures of wild-type *E. coli* were treated with ampicillin (8 MIC) or ampicillin plus 0.33 M, 16.7 mM or 333 µM lysine HCl for the indicated times after which samples were taken for CFU and percent survival determination. Data represent the mean from 3 biological replicates; error bars indicate standard deviations.

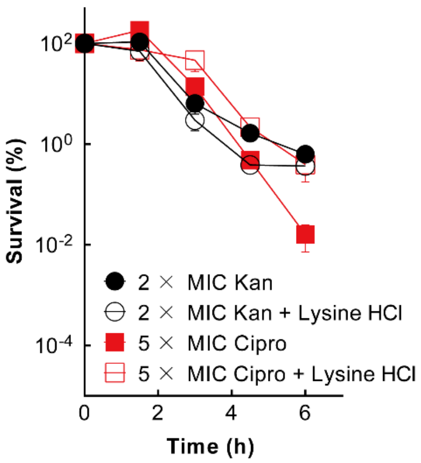


**Fig. S4. Lysine HCl exhibited little lethality enhancement with kanamycin or ciprofloxacin.** Exponentially growing cultures of wild type *E. coli* were treated with 2 MIC kanamycin (Kan) or 5 MIC ciprofloxacin (Cipro) in the presence/absence of 0.33 M (1/3 MIC) lysine HCl for the indicated times after which samples were processed as Fig. S1 for determination of percent survival. Data represent the mean from 3 biological replicates; error bars indicate standard deviations.

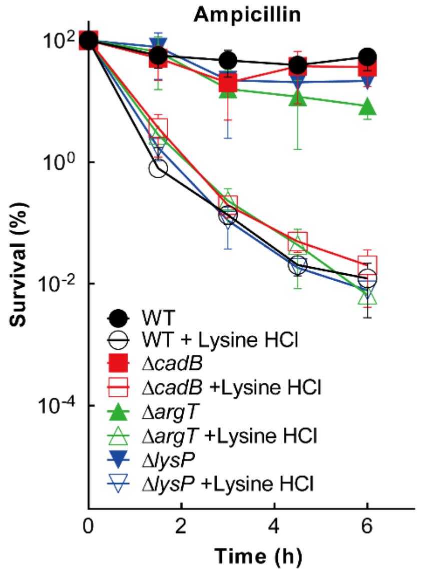


**Fig. S5. Transporter deficiency does not affect lysine HCl enhancement of ampicillin lethality.** Exponentially growing cultures of wild type, Δ*cadB*, Δ*lysP*, and Δ*argT* mutant cells were treated with 1 MIC ampicillin in the presence/absence of 1/3 MIC lysine HCl for the indicated times after which samples were processed as in Fig. S1 for determination of percent survival. Data represent the mean from 3 biological replicates; error bars indicate standard deviations.

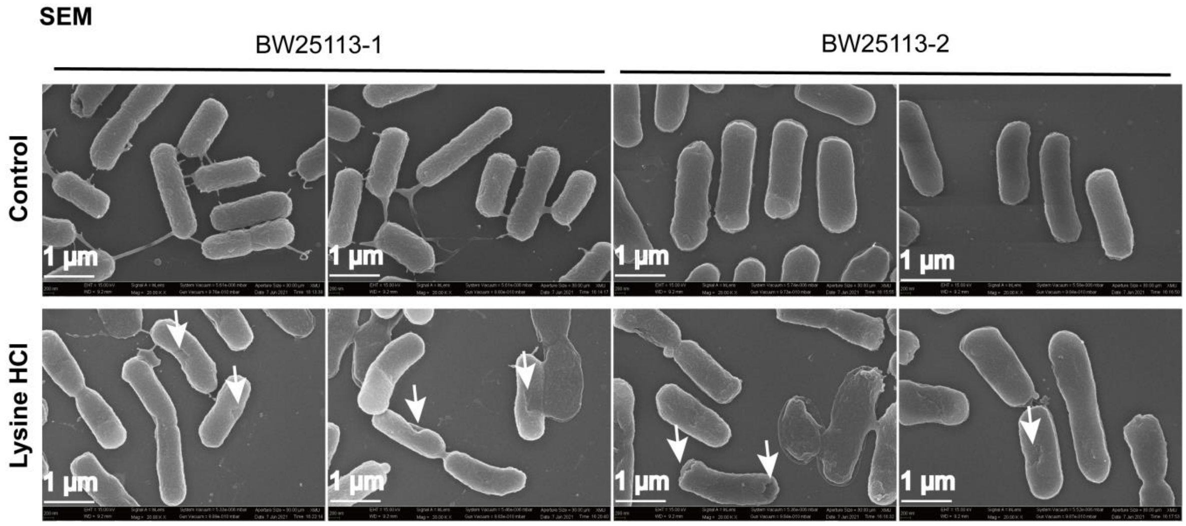


**Fig. S6. Lysine HCl damages bacterial cell surface.** Exponentially growing cultures of wild-type *E. coli* were treated/untreated with 0.33 M lysine HCl for 1.5 h before they were subjected to scanning electron microscopy. Arrows point to areas of cell surface damage. Similar data were obtained from 2 biological replicates. (BW25113-1 and BW25113-2); two technical repeats were shown for each biological replicate.

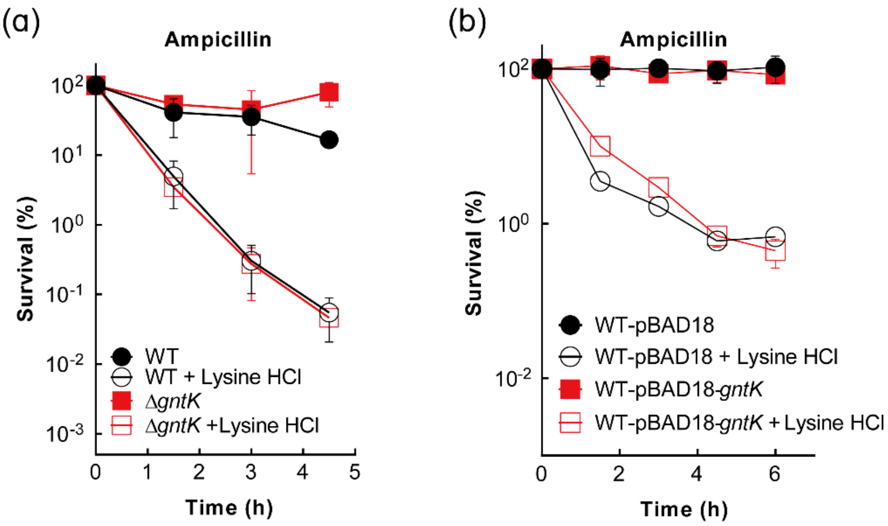


**Fig. S7. Neither a deficiency nor overexpression of *gntK* affects lysine stimulation of β-lactam killing. (a)** Effect of a *gntK* deficiency. Exponentially growing cultures of wild-type (WT) and a Δ*gntK* mutant were treated with 1/3 MIC lysine HCl and 1 MIC Amp for the indicated times after which samples were processed as in Fig. S1 for determination of percent survival. **(b)** Effect of *gntK* overexpression. Exponentially growing cultures of WT-pBAD18 and WT-pBAD18-*gntK* were pre-treated with 0.1% arabinose before ampicillin or an ampicillin-lysine HCl combination treatment and sample processing were carried out as in panel **a**. Data represent the mean from 3 biological replicates; error bars indicate standard deviations.

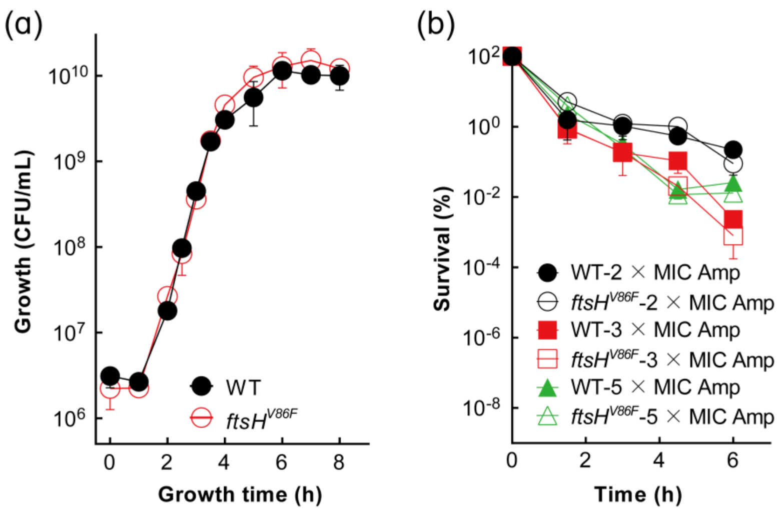


**Fig. S8. The *ftsH* V86F mutation affects neither bacterial growth nor ampicillin lethality.** **(a)** Effect on bacterial growth. Overnight cultures of wild-type and the *ftsH* V86F mutant were diluted in fresh LB broth by 2000-fold and then grown aerobically at 37°C for the indicated times before samples were taken for colony-forming unit determination as Fig. S1a. **(b)** Effect on ampicillin lethality. Exponentially growing cultures of *E. coli* wildtype (WT) and the *ftsH* V86F mutant were treated with 2, 3, or 5 MIC ampicillin for the indicated times before percent survival was determined as Fig. S1. Data represent the mean from 3 biological replicates; error bars indicate standard deviations.

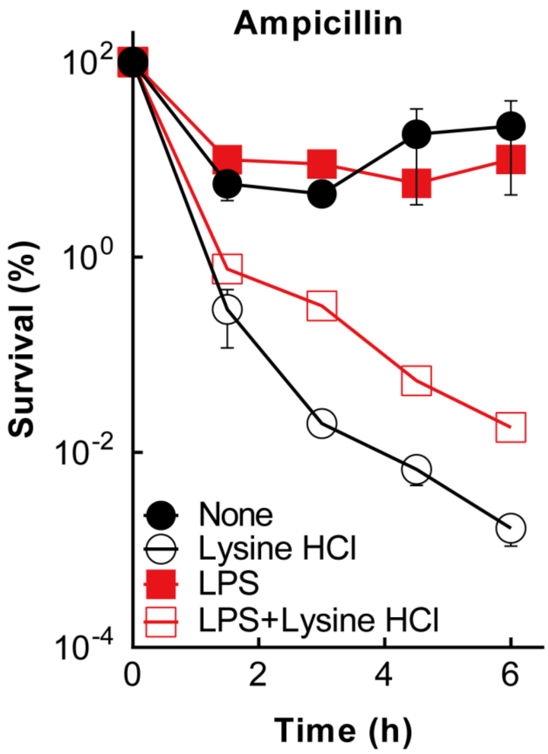


**Fig. S9. Pre-incubation with LPS reduced lysine HCl enhancement of ampicillin lethality.** Lysine HCl (0.33 M) were incubated with or without 0.66 mg/mL LPS at 37℃ for 30 min before the mixture, along with ampicillin (1 MIC), was added to exponentially growing cultures of wild-type *E. coli* for incubation. Samples were taken at the indicated times for CFU and percent survival determination. Data represent the mean from 3 biological replicates; error bars indicate standard deviations.

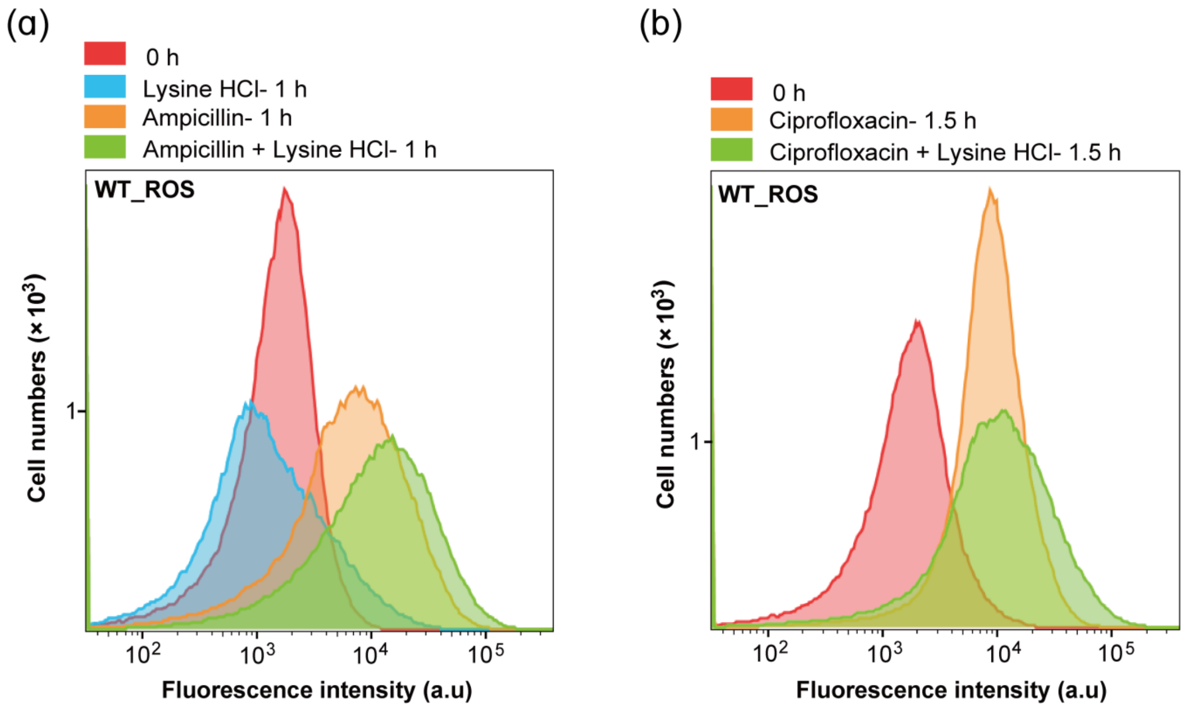


**Fig. S10. Measurement of intracellular ROS accumulation during lysine HCl-ampicillin or lysine-HCl-ciprofloxacin combination treatment.** Exponentially growing cultures of wild-type *E. coli* were pre-treated with 5 µM carboxy-H2DCFDA for 20 min and then exposed to 0.33 M lysine HCl, 1 MIC ampicillin, and a lysine HCl-ampicillin combination (a) or 5 MIC ciprofloxacin and a lysine HCl- ciprofloxacin combination (b) for the indicated times. Samples were then subjected for flow cytometry to assess fluorescence as an indicator of intracellular ROS level. Curves farther to the right indicate higher levels of ROS. Similar data were obtained from 3 biological replicates.

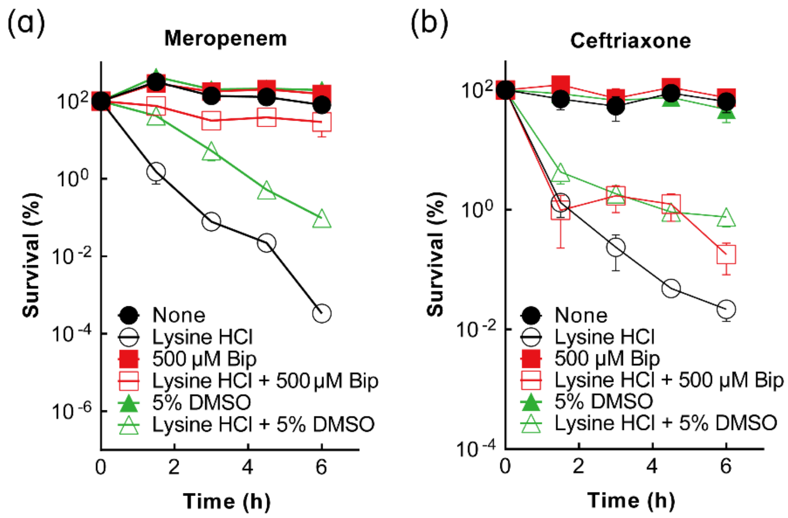


**Fig. S11. Protection from a lysine HCl-ampicillin combination-mediated killing by chemical suppression of ROS.** Exponentially growing cultures of wild-type *E. coli* were pre-treated with 5% DMSO or 500 μM bipyridyl for 20 min before they were treated with meropenem (2 MIC, **a**) or ceftriaxone (2 MIC, **b**) in the presence/absence of 1/3 MIC lysine HCl for the indicated times before percent survival was determined as Fig. S1. Data represent the mean from 3 biological replicates; error bars indicate standard deviations.

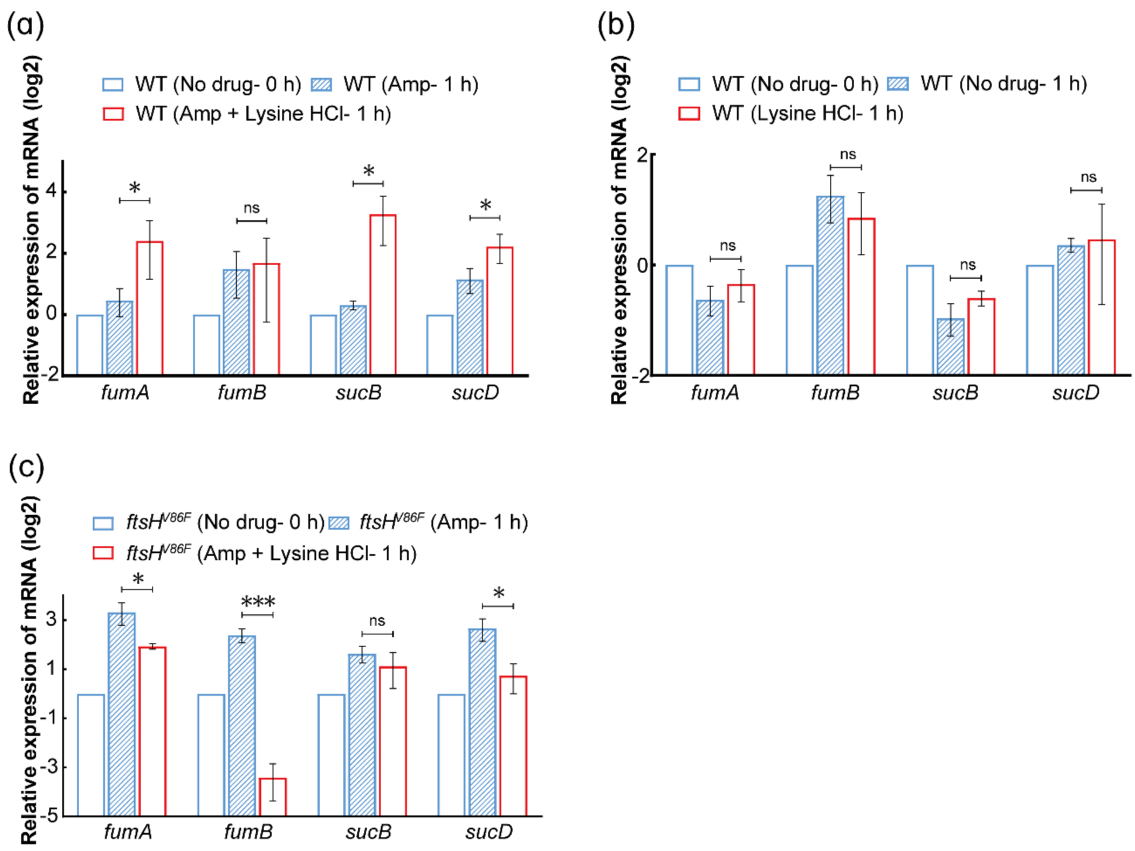


**Fig. S12. An *ftsH* V86F mutation eliminated the upregulation of several TCA cycle genes by the ampicillin-lysine HCl combination. (a)** Stimulation of ampicillin-induced TCA cycle gene expression by lysine HCl in wild-type cells. **(b)** Lysine HCl alone failed to stimulate TCA cycle gene expression. **(c)** Reversal of lysine HCl-stimulated, ampicillin-induced TCA cycle gene expression by an *ftsH* V86F mutation. Decreased expression is shown for genes participating in the TCA cycle of an *ftsH* V86F mutant (**c**), but not in wild-type cells **(a)** when exposed to a lysine HCl-ampicillin combination compared with ampicillin exposure alone. Exponentially growing cultures of wild-type (**a and b**) and the *ftsH* V86F mutant (**c**) were treated with 1 MIC ampicillin in the presence/absence of 1/3 MIC lysine HCl (0.33 M) for 0 or 1 h before total bacterial RNA was extracted, reverse transcribed, and amplified with primers encoding the indicated TCA cycle genes by PCR using the 16S rRNA gene as an internal control. The mRNA level of each gene of interest was normalized

to the 16S rRNA gene and that of the same gene of untreated WT or *ftsH* V86F mutant samples. Data represent the mean from 3 biological replicates; error bars indicate standard deviations. *, $P < 0.05$; ***, $P < 0.001$.

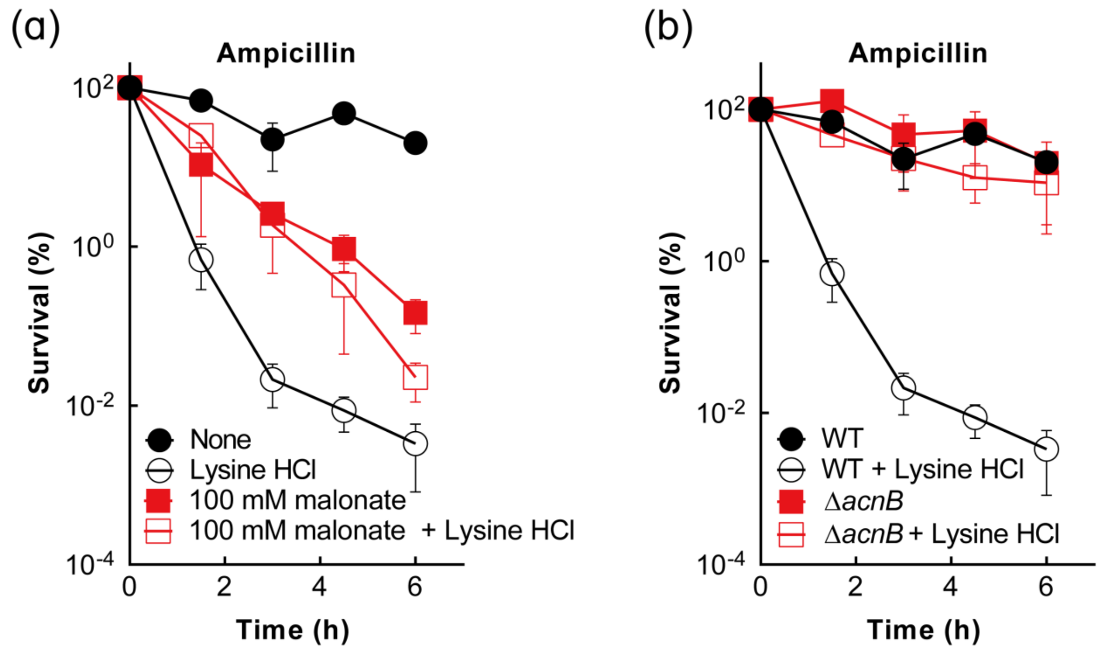


**Fig. S13. Suppression of lysine HCl enhancement of β-lactam lethality by malonate addition and by a deficiency in *acnB*. (a)** Exponentially growing cultures of wild-type *E. coli* were pre-treated with 100 mM malonate for 20 min before addition of ampicillin (1 MIC) or ampicillin plus lysine HCl (0.33 M) for the indicated times after which samples were taken for CFU and percent survival determination. **(b)** Exponentially growing cultures of wild-type *E. coli* and Δ*acnB* mutant were treated with ampicillin (1 MIC) or ampicillin plus lysine HCl (0.33 M) for the indicated times and samples were processed as panel a. Data represent the mean from 3 biological replicates; error bars indicate standard deviations.

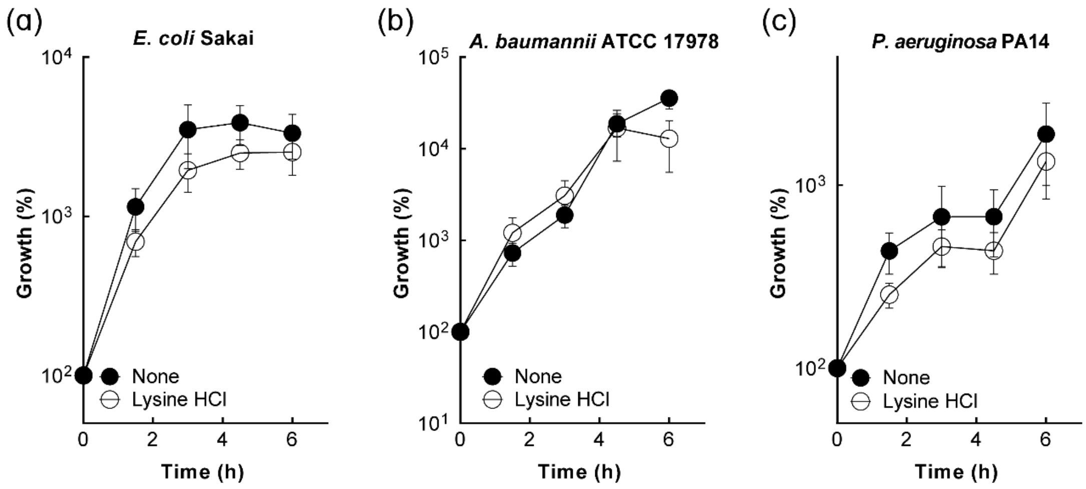


**Fig. S14. Little growth inhibition of pathogenic Gram-negative bacteria treated with the lysine HCl concentration used for lethality enhancement.** Overnight cultures of *E. coli* Sakai (**a**), *A. baumannii* ATCC17978 (**b**), or *P. aeruginosa* PA14 (**c**) were diluted 100-fold into fresh LB broth and incubated aerobically at 37°C for 1 h before lysine HCl (1/3 MIC) was added to the culture. Samples were taken at the indicated times for colony-forming unit determination and percent survival was calculated as in Fig. S1. Data represent the mean from 3 biological replicates; error bars indicate standard deviations.

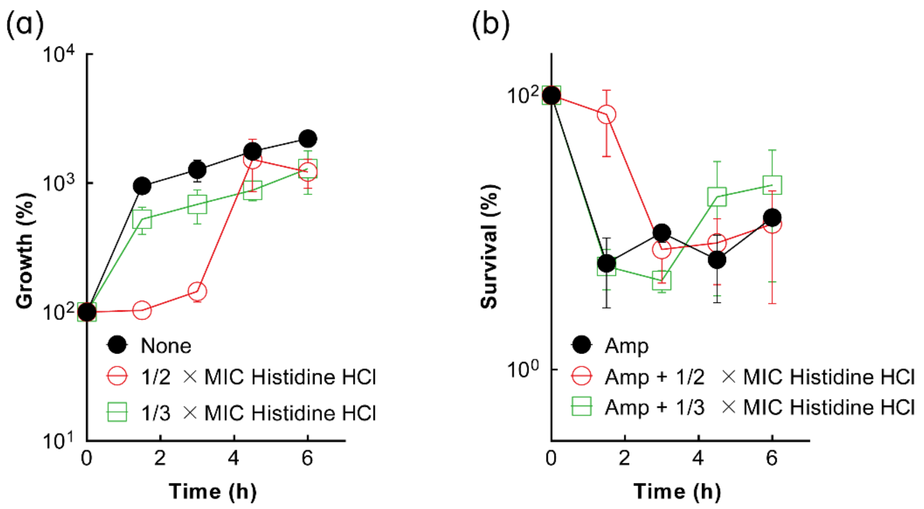


**Fig. S15. Little Lethality enhancement of β-lactam-mediated killing by histidine HCl.** **(a)** Effect of histidine concentration on growth. Overnight cultures of wild type *E. coli* were diluted 100-fold into fresh LB medium and incubated aerobically at 37°C or 1 h before histidine HCl (0, 1/3, 1/2 MIC) was added for continued incubation. Samples were taken at the indicated times for determination of colony-forming units and percent survival/growth calculation. **(b)** Effect of histidine HCl concentration on ampicillin-mediated killing. Exponentially growing cultures of wild type *E. coli* were treated with ampicillin (1 MIC) in the presence of 0, 1/3, or 1/2 MIC of histidine HCl for the indicated times before samples were processed as Fig. S1 for percent survival determination. Data represent the mean from 3 biological replicates; error bars indicate standard deviations.

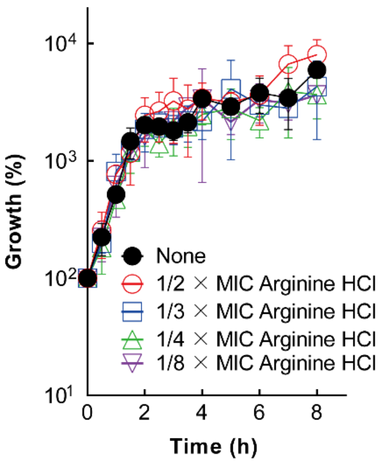


**Fig. S16. Effect of arginine HCl concentration on *E. coli* growth.** Overnight cultures of wild-type *E. coli* were diluted 1:100 into fresh LB broth and grown for 1 h before the indicated concentrations of arginine HCl were added. Cultures were incubated for the indicated times at which samples were taken and processed as Fig. S1 for colony-forming unit and percent growth/survival determination. Data represent the mean from 3 biological replicates; error bars indicate standard deviations.

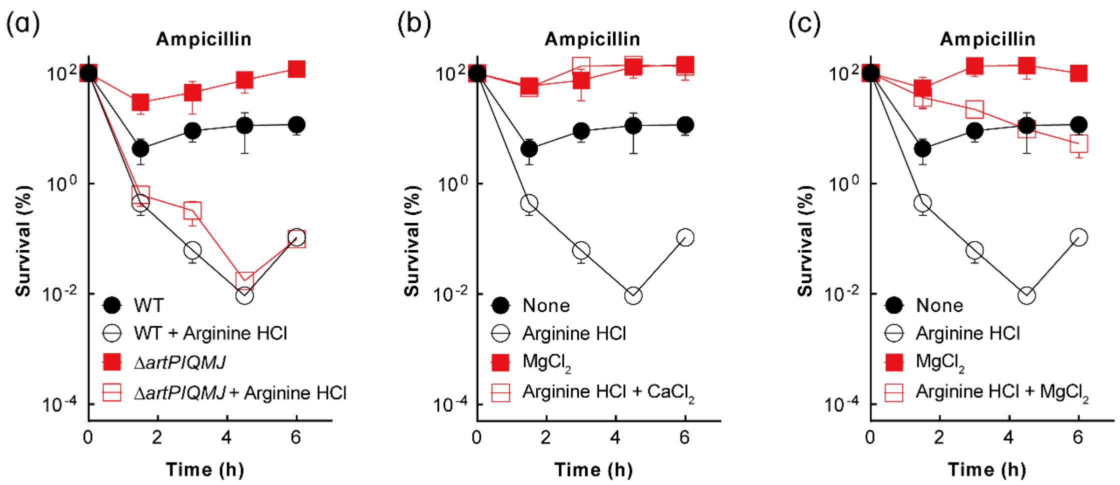


**Fig. S17. Arginine HCl enhances ampicillin-mediated killing by destabilizing outer membrane. (a)** Cellular uptake of arginine HCl is not required for enhancement of ampicillin-mediated killing. Exponentially growing cultures of wild-type and Δ*artPIQMJ* mutant *E. coli* were treated with 1 MIC ampicillin in the presence/absence of 1/2 MIC arginine HCl for the indicated times before percent survival was determined as in Fig. S1 **(b and c)**. Stabilization of outer membrane by divalent ions eliminated arginine HCl-mediated lethality enhancement of ampicillin. Exponentially growing cultures of wild-type *E. coli* were treated with 1 MIC ampicillin plus/minus 1/2 MIC arginine HCl in the presence/absence of 20 mM $CaCl_2$ **(b)** or 62.5 mM $MgCl_2$ **(c)** for the indicated times after which samples were processed as Fig. S1. Data represent the mean from 3 biological replicates; error bars indicate standard deviations.

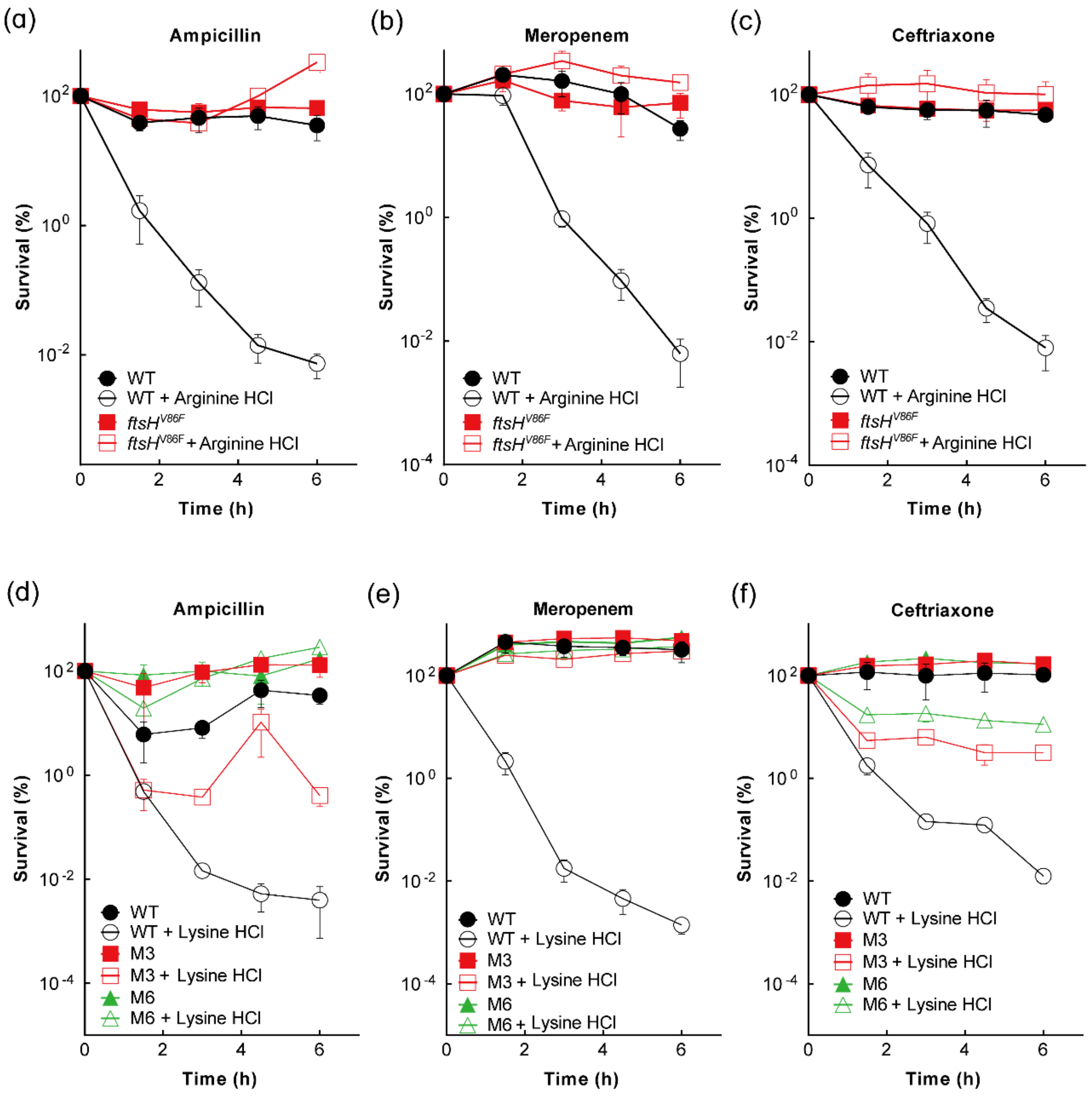


**Fig. S18. Cross tolerance to enhancement of β-lactam lethality between mutants selected against lysine HCl- and arginine HCl-ampicillin combinations. (a-c)** The *ftsH* V86F mutant, obtained from lysine HCl-ampicillin enrichment, suppressed arginine HCl-stimulated β-lactam-mediated killing. Exponentially growing cultures of wild-type (WT) and the *ftsH* V86F mutant were treated with 1 MIC ampicillin **(a)**, 2 MIC meropenem **(b)** or 2 MIC ceftriaxone **(c)** in the presence/absence of 1/2 MIC arginine HCl for the indicated times. Samples were then taken, diluted, and plated on drug-free agar for CFU and percent survival determination as Fig. S1. **(d-f)** The M3 and M6 mutants, selected for tolerance to arginine

HCl stimulation of ampicillin-mediated killing conferred cross-tolerance to lysine HCl enhancement of β-lactam lethality. Exponentially growing cultures of wild-type, M3 and M6 mutants were treated with 1 MIC ampicillin **(d)**, 2 MIC meropenem **(e)** or 2 MIC ceftriaxone **(f)** in the presence/absence of 1/3 MIC lysine HCl for the indicated times. Samples were then taken, diluted, and plated on drug-free agar for CFU and percent survival determination as in Fig. S1. Data represent the mean from 3 biological replicates; error bars indicate standard deviations.

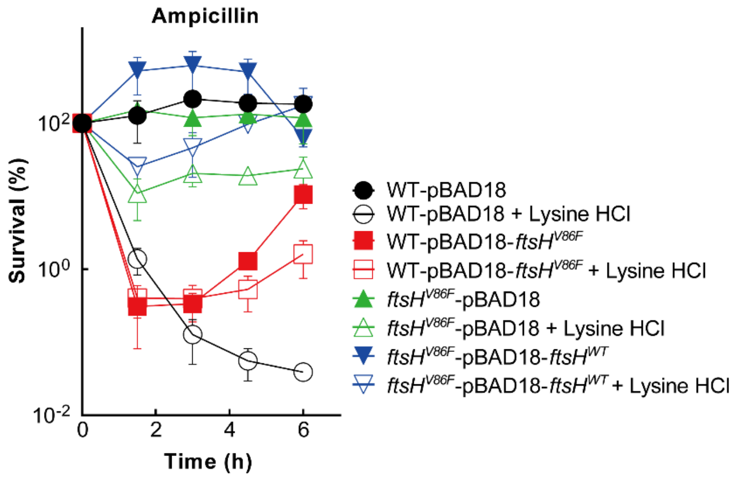


**Fig. S19. An *ftsH* V86F mutant allele confers gain-of-function phenotype.** Over-expression of *ftsH*$^{WT}$ from a plasmid in *ftsH* V86F mutant failed to reverse *ftsH* V86F-mediated tolerance to enhanced β-lactam lethality and over-expression of *ftsH*$^{V86F}$ from a plasmid in wild-type strain eliminated lysine-mediated enhancement of β-lactam lethality. Exponentially growing cultures of *E. coli* wild-type strain harboring pBAD18 or pBAD18-*ftsH*$^{V86F}$, and an *ftsH* V86F mutant strain harboring pBAD18 or pBAD18-*ftsH*$^{WT}$ were treated with ampicillin alone or a lysine HCl-ampicillin combination for the indicated times before samples were taken, diluted, and plated for percent survival determination. Data represent the mean from 3 biological replicates; error bars indicate standard deviations.

**Table S1. Bacterial strains and MIC for ampicillin, lysine HCl, arginine HCl.**

| Strain information | | | MIC | | |
|---|---|---|---|---|---|
| Strain number | Relevant genotype | Source and description | Ampicillin (μg/mL) | Lysine[a] (M) | Arginine[a] (M) |
| 1 | Wild type | *E. coli* BW25113 | 4 | 1 | 0.375 |
| 2 | Δ*cadB::kan*, $kan^R$ | Keio collection, JW4092 | 4 | 1 | ND[c] |
| 3 | Δ*lysP::kan*, $kan^R$ | Keio collection, JW2143 | 4 | 1 | ND |
| 4 | Δ*argT::kan*, $kan^R$ | Keio collection, JW2307 | 4 | 1 | ND |
| 5 | Δ*cadB*Δ*lysP*Δ*argT* | This work, constructed by CRISPR-cas9 | 4 | 1 | ND |
| 6 | LYS1 | This study, Lysine-ampicillin combination-tolerant mutant 1 | 4 | 0.75 | ND |
| 7 | LYS3 | This study, Lysine-ampicillin combination-tolerant mutant 3 | 4 | 0.75 | ND |
| 8 | *ftsH* V86F | This work, constructed by CRISPR-cas9 | 4 | 0.75 | 0.375 |
| 9 | $ftsH^{V86F}::ftsH^{WT}$ | This work, constructed by CRISPR-cas9 | 4 | 1 | ND |
| 10 | Δ*gntK::kan*, $kan^R$ | Keio collection, JW3400 | 4 | 1 | ND |
| 11 | WT-pBAD18 | Wild-type strain transformed with pBAD18 | 4 | 1 | ND |
| 12 | WT-pBAD18-*gntK* | Wild-type strain transformed with pBAD18-*gntK* | 4 | 1 | ND |

| | | | | | |
|---|---|---|---|---|---|
| 13 | *ftsH*$^{V86F}$ -pBAD18 | *ftsH*$^{V86F}$ strain transformed with pBAD18 | 4 | 0.75 | ND |
| 14 | *ftsH*$^{V86F}$-pBAD18-*lpxC* | *ftsH*$^{V86F}$ strain transformed with pBAD18-*lpxC* | 4 | 1 | ND |
| 15 | *lpxC* H19T | This work, constructed by CRISPR-cas9 | 4 | 0.5 | ND |
| 16 | *ftsH*$^{V86F}$*lpxC*$^{H19T}$ | This work, constructed by CRISPR-cas9 | 1 | 0.375 | ND |
| 17 | Δ*artPIQMJ* | This work, constructed by CRISPR-cas9 | 4 | ND | 0.375 |
| 18 | M3 | This study, arginine-ampicillin combination-tolerant mutant 3 | 4 | 0.75 | 0.375 |
| 19 | M6 | This study, arginine-ampicillin combination-tolerant mutant 6 | 4 | 0.75 | 0.375 |
| 20 | WT-pBAD18-*ftsH*$^{V86F}$ | Wild-type strain transformed with pBAD18-*ftsH*$^{V86F}$ | 3 | 0.75 | ND |
| 21 | *ftsH*$^{V86F}$-pBAD18-*ftsH*$^{WT}$ | *ftsH*$^{V86F}$ strain transformed with pBAD18- *ftsH*$^{WT}$ | 3 | 0.75 | ND |
| 22 | Δ*acnB::kan, kan*$^{R}$ | Keio collection, JW0114 | 4 | 1 | ND |

[a]Lysine HCl; arginine HCl

[b]kan, kanamycin

[c]Not determined

**Table S2. Minimal Inhibitory Concentrations (MIC) of compounds tested**

| Chemical | Strains (MIC (µg/mL)) | | | | | | |
|---|---|---|---|---|---|---|---|
| | WT[a] | *ftsH* V86F | $ftsH^{V86F}::ftsH^{WT}$ | LYS1 | LYS3 | M3 | M6 |
| Meropenem | 0.04 | 0.04 | 0.04 | 0.04 | 0.04 | 0.04 | 0.04 |
| Ceftriaxone | 0.04 | 0.04 | 0.04 | 0.04 | 0.04 | 0.04 | 0.04 |
| $MgCl_2$ | 0.25 M[b] | ND[c] | ND | ND | ND | ND | ND |
| $CaCl_2$ | 1.6 M | ND | ND | ND | ND | ND | ND |
| Histidine HCl | 0.375 M | ND | ND | ND | ND | ND | ND |
| Malonate | 800 mM | ND | ND | ND | ND | ND | ND |
| Ampicillin + 100 mM malonate | 3 | ND | ND | ND | ND | ND | ND |
| + 0.66 mg/mL LPS | 4 | ND | ND | ND | ND | ND | ND |

[a]WT, wild type; [b]M, MIC in molar concentration; [c]ND, not determined;

**Table S3. Mutational information of drug combination-tolerant mutants**

| Mutant | Mutant gene | Base change | Amino acid change | Gene product |
|---|---|---|---|---|
| M3 | *ftsH* | T137C | Val46Ala | ATP-dependent zinc metalloprotease FtsH |
| M6 | *ftsH* | T122G | Val41Gly | ATP-dependent zinc metalloprotease FtsH |

## Table S4. Primers used in the study

| Primer name | Purpose | Nucleotide sequence of primers (5'-3') |
|---|---|---|
| Keio-check-R | Downstream primer for Keio mutant strain confirmation | AGTCATAGCCGAATAGCCTC |
| *cadB*-keio-F | Downstream primer for Δ*cadB::kan* confirmation | ACTTGCGATCCTGATGTG |
| *gntK*-keio-F | Downstream primer for Δ*gntK::kan* confirmation | ATTGGTCAGGTGATGGAG |
| *argT*-keio-F | Amplification of Δ*argT::FRT* homologous fragment from Δ*argT::FRT* template for CRISPR-based deletion | CCGCTATACCTGCTATCTTC |
| *argT*-R | | AGAACCAGAGAGAGCGATAG |
| *lysP*-keio-F | Amplification of Δ*lysP::FRT* homologous fragment from Δ*lysP::FRT* template for CRISPR-based deletion | TGGTGCTGTGTCGATTTC |
| *lysP*-R | | CTCACATCTTCTTCACGTCC |
| FRT-sgRNA-F | Construction of sgRNA targeting the FRT sequence of Keio mutants for backcrossing mutant alleles into the corresponding Keio mutant strain | TCCTAGGTATAATACTAGTTCCTATTCTCTAGAAAGTATGTTTTAGAGCTAGAAATAGC (Spe I) |
| FRT-sgRNA-R | | AACTGCAGTTCAAAAAAAGCACCGACTCGG (Pst I) |
| *ftsH*$^{V86F}$-sgRNA-F | Amplification of *ftsH*$^{V86F}$-sgRNA for CRISPER-based mutant construction | GGACTAGTCCTGTTGACCAAGAACGTCAGTTTTAGAGCTAGAAATAGC (Spe I) |
| sgRNA-R | | CTCAAAAAAAGCACCGACTCGG |

| | | |
|---|---|---|
| *ftsH*$^{V86F}$-up-F | Primers for PCR amplification of *ftsH*$^{V86F}$ upstream homologous template for CRISPR-based allelic exchange | CGAGTCGGTGCTTTTTTG AGTTGTAGTGAAGGTGTTC CAG |
| *ftsH*$^{V86F}$-up-R | | GGTTCACCGACAACTTTGA AGTTCTTGGTCAACAGGTT ATC |
| *ftsH*$^{V86F}$-down-F | Primers for PCR amplification of *ftsH*$^{V86F}$ downstream homologous template for CRISPR-based allelic exchange | TCAAAGTTGTCGGTGAACC GCCT |
| *ftsH*$^{V86F}$-down-R | | GAAGATCTCGCTTTCTTCGC CTGTTC (Bgl II) |
| *ftsH*$^{V86F}$-check-F | Upstream primer for *ftsH*$^{V86F}$, *ftsH*$^{WT}$ confirmation | TACTCTGGCTGGTCATTG |
| *ftsH*$^{V86F}$-check-R | Downstream primer for *ftsH*$^{V86F}$, *ftsH*$^{WT}$ confirmation | ACCATCAAGACGCCTTTC |
| *ftsH*$^{WT}$-sgRNA-F | Preparation of sgRNA for restoring wild-type *ftsH* in *ftsH*$^{V86F}$ mutant by CRISPR-based knock-in | GGACTAGTACCGACAACTT TGAAGTTCTGTTTTAGAGCT AGAAATAGC (Spe I) |
| *ftsH*$^{WT}$-up-R | Primers for PCR amplification of wild-type *ftsH* upstream homologous template for restoring *ftsH*$^{V86F}$ back to wild-type *ftsH* by CRISPR | GACAACCTTGACGTTCTTA GTCAACAGGTTATCCAGT |
| *ftsH*$^{WT}$- down -F | Primers for PCR amplification of wild-type *ftsH* downstream homologous template for restoring *ftsH*$^{V86F}$ | CTAAGAACGTCAAGGTTGT C |

| | | |
|---|---|---|
| | back to wild-type *ftsH* by CRISPR | |
| *gntK-F* | Primers for constructing recombinant pBAD18-*gntK* for overexpression of *gntK* | GGGGTACCTTTAACTTTAAGAAGGAGATATACATTTGAGCACGACTAACCATGATC (Kpn I) |
| *gntK-R* | | GCTCTAGATTATTTGCCTTTTTTAATAACC (Xba I) |
| *lpxC-F* | Primers for constructing recombinant pBAD18-*lpxC* for overexpression of *lpxC* | GGGGTACCTTTAACTTTAAGAAGGAGATATACATATGATCAAACAAAGGACAC (Kpn I) |
| *lpxC-R* | | GCTCTAGATCCACAGCACGAATGAAG (Xba I) |
| *lpxC*$^{H19T}$-sgRNA -F | Amplification of *lpxC*$^{H19T}$-sgRNA for CRISPR-based mutant construction | GGACTAGTCGTCAGGGTGACTTTCTTGCGTTTTAGAGCTAGAAATAGC(SpeI) |
| *lpxC*$^{H19T}$-UP-F | Primers for PCR amplification of *lpxC*$^{H19T}$ upstream homologous template for CRISPR-based allelic exchange | CGAGTCGGTGCTTTTTTTGAGCGACTGTGGTTATCGGTAC |
| *lpxC*$^{H19T}$-UP-R | | CTTGCCTGTATATAAACCGACACCCGTCGCCTG |
| *lpxC*$^{H19T}$-down-F | Primers for PCR amplification of *lpxC*$^{H19T}$ downstream homologous template for CRISPR-based allelic exchange | TTATATACAGGCAAGAAAGTCACCCTGAC |
| *lpxC*$^{H19T}$-down-R | | GAAGATCTCTGGAATCAATAGCCGGATG (Bgl II) |
| *lpxC*$^{H19T}$-check-F | Upstream primer for *lpxC*$^{H19T}$ confirmation | ATCCGTGCATTTGCTTCC |

| | | |
|---|---|---|
| $lpxC^{H19T}$-check-R | Downstream primer for $lpxC^{H19T}$ confirmation | CCACACATGAACAAGTCACC |
| *fumA*-F | Primers used for *fumA* mRNA level determination by RT-PCR | CGAAGATCAACCGTCAGGGG |
| *fumA*-R | | GCCGTTAAGCGATAAGCGTG |
| *fumB*-F | Primers used for *fumB* mRNA level determination by RT-PCR | GCGGTGATGAAGAAACGCTG |
| *fumB*-R | | CCTTTCGCAACGCAAAGGAA |
| *SucB*-F | Primers used for *sucB* mRNA level determination by RT-PCR | GGAAGCGAAAAACTCCACC |
| *SucB*-R | | GAAGCGTTCACTTCCGGGTA |
| *SucD*-F | Primers used for *sucD* mRNA level determination by RT-PCR | TTTCGGTCAGTCGACCTGTG |
| *SucD*-R | | CAACTGGCTTGGTAACGTG |
| *16S*-F | Primers used for *16S* mRNA level determination by RT-PCR | CTTACGACCAGGGCTACACAC |
| *16S*-R | | CGGACTACGACGCACTTTATG |
| *ftsH -F* | Primers for constructing recombinant pBAD18-$ftsH^{WT}$/-$ftsH^{V86F}$ for overexpression of $ftsH^{WT}$ *or* $ftsH^{V86F}$ | CGGAATTCTTTAACTTTAAGAAGGAGATATACATatgGCGAAAAACCTAATACTC (Kpn I) |
| *ftsH-R* | | GCTCTAGATTTCATGATGTTATCCCTGG (Xba I) |
| | | |
| **plasmids** | **Characteristics**[a] | **Source or reference** |
| pCP20 | Plasmid containing yeast Flp recombinase for antibiotic marker removal; $Amp^R$, $Cm^R$ | (1), (2) |

| | | |
|---|---|---|
| pCas9 | Kan$^R$, *repA*101ts, *lac*Iq-Ptrc fragment, sg-RNA-pMBI, λ-Red recombinase gene (exo bet gam paraB) | (3) |
| pTargetF | aadA (spec$^R$), pMB1, pj23119 (synthetic promoter)-N20-sgRNA | (3) |
| pTargetF-sgRNA-FRT | spec$^R$, sgRNA with an N20 sequence targeting the *FRT* region of marker-less Keio library mutant. | This study |
| pBAD18 | *rep*pBR322*bla* Kan$^R$ | (4) |
| pBAD18-*gntK* | Coding sequence *of gntK* cloned into pBAD18 | This study |
| pBAD18-*lpxC* | Coding sequence *of lpxC* cloned into pBAD18 | This study |
| pTargetF -sgRNA-*ftsH*$^{V86F}$ | spec$^R$, sgRNA with an N20 sequence targeting the *ftsH* gene of the wildtype strain, also containing homologous template for *ftsH* allelic exchange | This study |
| pTargetF -sgRNA-*ftsH*$^{WT}$ | spec$^R$, sgRNA with an N20 sequence targeting the *ftsH* gene of the *ftsH*$^{V86F}$ strain, also containing homologous | This study |

| | | |
|---|---|---|
| | template for *ftsH* allelic exchange | |
| pTargetF -sgRNA-*lpxC*$^{H19T}$ | spec$^{R}$, sgRNA with an N20 sequence targeting the *lpxC* gene of the wildtype strain, also containing homologous template for *lpxC* allelic exchange | This study |
| pBAD18- *ftsH*$^{WT}$ | Coding sequence *of ftsH*$^{WT}$ cloned into pBAD18 | This study |
| pBAD18- *ftsH*$^{V86F}$ | Coding sequence *of ftsH*$^{V86F}$ cloned into pBAD18 | This study |

## Supplementary references


1. Cherepanov PP, Wackernagel W. 1995. Gene disruption in *Escherichia coli*: TcR and KmR cassettes with the option of Flp-catalyzed excision of the antibiotic-resistance determinant. Gene 158:9-14. https://doi.org/10.1016/0378-1119(95)00193-a.
2. Baba T, Ara T, Hasegawa M, Takai Y, Okumura Y, Baba M, Datsenko KA, Tomita M, Wanner BL, Mori H. 2006. Construction of *Escherichia coli* K-12 in-frame, single-gene knockout mutants: the Keio collection. Mol Syst Biol 2:2006 0008. https://doi.org/10.1038/msb4100050.
3. Jiang Y, Chen B, Duan C, Sun B, Yang J, Yang S. 2015. Multigene editing in the *Escherichia coli* genome via the CRISPR-Cas9 system. Appl Environ Microbiol 81:2506-14. https://doi.org/10.1128/AEM.04023-14.

4. Guzman LM, Belin D, Carson MJ, Beckwith J. 1995. Tight regulation, modulation, and high-level expression by vectors containing the arabinose PBAD promoter. J Bacteriol 177:4121-30. https://doi.org/10.1128/jb.177.14.4121-4130.1995.
